# Association between acetaminophen metabolites and *CYP2E1* DNA methylation level in neonate cord blood in the Boston Birth Cohort

**DOI:** 10.1186/s13148-023-01551-4

**Published:** 2023-08-18

**Authors:** Yijun Li, Xiumei Hong, Liming Liang, Xiaobin Wang, Christine Ladd-Acosta

**Affiliations:** 1 Department of Epidemiology, Johns Hopkins Bloomberg School of Public Health, 615 N. Wolfe Street W6509, Baltimore, MD 21205 USA; 2 Department of Epidemiology, Harvard T.H. Chan School of Public Health, Boston, MA USA; 3 Center on the Early Life Origins of Disease, Department of Population, Family and Reproductive Health, Johns Hopkins Bloomberg School of Public Health, Baltimore, MD USA; 4 Department of Pediatrics, Johns Hopkins School of Medicine, Baltimore, MD USA

**Keywords:** Perinatal acetaminophen, DNA methylation, *CYP2E1*

## Abstract

**Background:**

Acetaminophen is a commonly used medication by pregnant women and is known to cross the placenta. However, little is known about the biological mechanisms that regulate acetaminophen in the developing offspring. Cytochrome 2E1 (CYP2E1) is the primary enzyme responsible for the conversion of acetaminophen to its toxic metabolite. Ex vivo studies have shown that the *CYP2E1* gene expression in human fetal liver and placenta is largely controlled by DNA methylation (DNAm) at CpG sites located in the gene body of *CYP2E1* at the 5’ end. To date, no population studies have examined the association between acetaminophen metabolite and fetal DNAm of *CYP2E1* at birth.

**Methods:**

We utilized data from the Boston Birth Cohort (BBC) which represents an urban, low-income, racially and ethnically diverse population in Boston, Massachusetts. Acetaminophen metabolites were measured in the cord plasma of newborns enrolled in BBC between 2003 and 2013 using liquid chromatography-tandem mass spectrometry. DNAm at 28 CpG sites of *CYP2E1* was measured by Illumina Infinium MethylationEPIC BeadChip. We used linear regression to identify differentially methylated CpG sites and the “DiffVar” method to identify differences in methylation variation associated with the detection of acetaminophen, adjusting for cell heterogeneity and batch effects. The false discovery rate (FDR) was calculated to account for multiple comparisons.

**Results:**

Among the 570 newborns included in this study, 96 (17%) had detectable acetaminophen in cord plasma. We identified 7 differentially methylated CpGs (FDR < 0.05) associated with the detection of acetaminophen and additional 4 CpGs showing a difference in the variation of methylation (FDR < 0.05). These CpGs were all located in the gene body of *CYP2E1* at the 5’ end and had a 3–6% lower average methylation level among participants with detectable acetaminophen compared to participants without. The CpG sites we identified overlap with previously identified DNase hypersensitivity and open chromatin regions in the ENCODE project, suggesting potential regulatory functions.

**Conclusions:**

In a US birth cohort, we found detection of cord biomarkers of acetaminophen was associated with DNAm level of *CYP2E1* in cord blood. Our findings suggest that DNA methylation of *CYP2E1* may be an important regulator of acetaminophen levels in newborns.

**Supplementary Information:**

The online version contains supplementary material available at 10.1186/s13148-023-01551-4.

## Background

Acetaminophen, also known as paracetamol, is the most widely used over-the-counter analgesic and antipyretic medication in pregnant women worldwide. In the USA, over 65% of pregnant women reported any use of acetaminophen during pregnancy, and 48% reported the use of acetaminophen in the third trimester [1]. Existing literature has shown transplacental passage of acetaminophen and its metabolites to the developing offspring after maternal use [2–4]. However, little is known about the biological processes that regulate acetaminophen levels in the offspring.

**Fig. 1:**
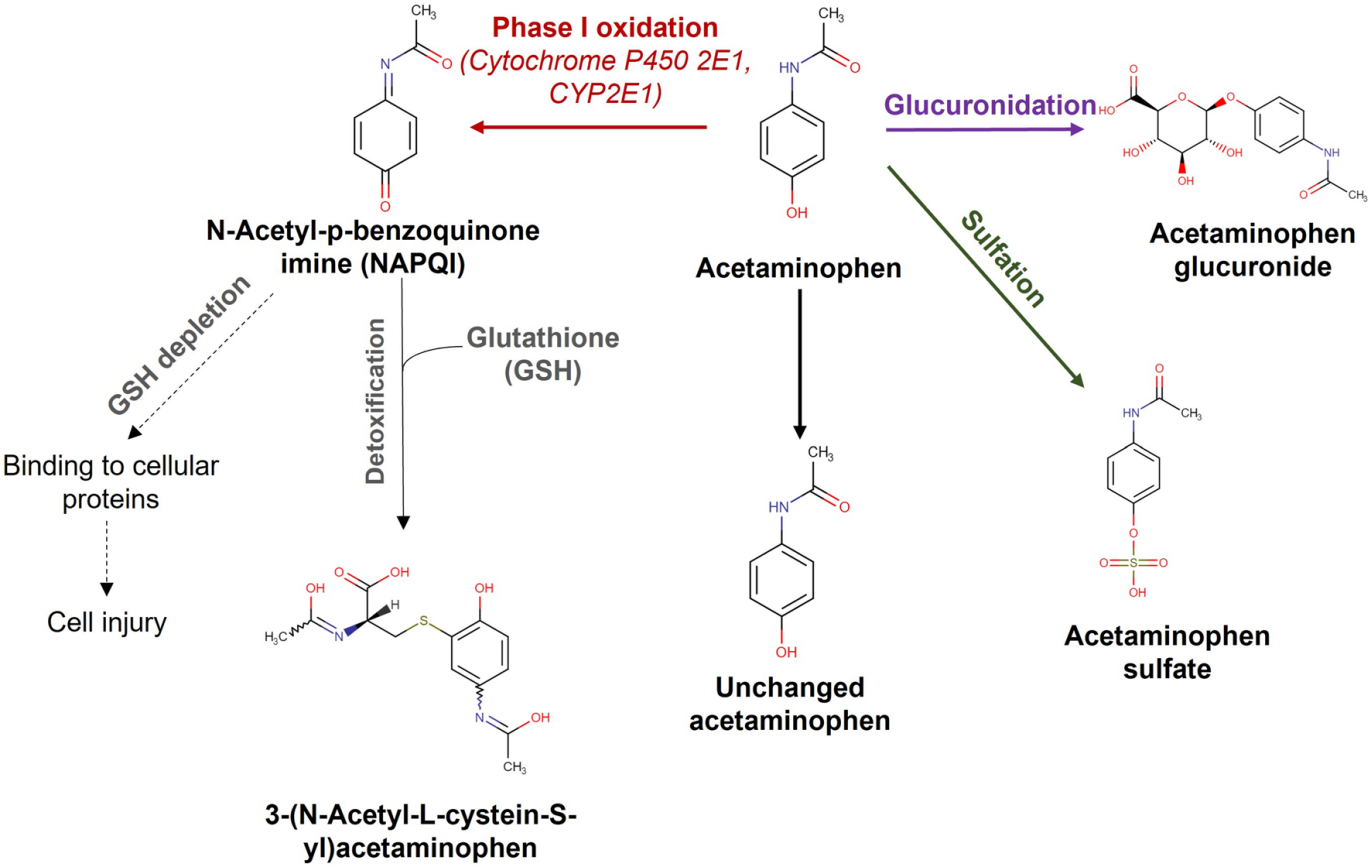
Pathways for acetaminophen metabolism. Acetaminophen metabolizes in liver through three main pathways: glucuronidation (shown in purple), sulfation (shown in green) and phase I oxidation (shown in red). Cytochrome P450 2E1 (*CYP2E1*) is the primary enzyme responsible for the bioconversion of acetaminophen to *N*-acetyl-*p*-benzoquinone imine (NAPQI) through the Phase I oxidation pathway. The chemical structures of the metabolites were obtained from The Human Metabolome Database (HMDB) (http://hmdb.ca)

Acetaminophen metabolism mainly occurs in the liver through the pathways illustrated in Fig. 1. Approximately 85% of acetaminophen conjugates with glucuronic and sulfuric acid, and forms non-toxic metabolites (acetaminophen glucuronide and acetaminophen sulfate, respectively) [5, 6]. A small proportion of acetaminophen (up to 5%) remains unchanged and excreted by urine [5, 6]. Up to 10% of acetaminophen undergoes phase I oxidation and forms *N*-acetyl-*p*-benzoquinone imine (NAPQI), a toxic metabolite that can cause cell injury [5, 6]. Cytochrome 2E1 (CYP2E1), encoded by the *CYP2E1* gene, is the primary enzyme responsible for the metabolic conversion of acetaminophen to NAPQI [5, 6]. In an experimental study, *CYP2E1* knockout mice were considerably less sensitive to the hepatotoxic effects of acetaminophen [7], suggesting a potential positive correlation between *CYP2E1* expression and acetaminophen. Multiple ex vivo studies have shown that the expression of *CYP2E1* in developing human tissues, including fetal liver [8, 9] and placenta [10], is primarily controlled by DNA methylation at CpG sites located in the gene body of *CYP2E1* at the 5’ end.

Given the central role of CYP2E1 in acetaminophen toxicity and the well-established evidence for the methylation regulation of *CYP2E1* expression, we sought to examine whether perinatal exposure to acetaminophen is associated with DNA methylation level at the *CYP2E1* gene in cord blood. We use unified DNA methylation and empiric metabolite measures of acetaminophen at the time of birth to facilitate the evaluation of the specific hypothesis that DNA methylation at CpG sites located in the gene body of *CYP2E1* at the 5’ end is associated with acetaminophen metabolites levels in the cord blood of newborns from a population-based cohort.

## Results

### Detection of acetaminophen biomarkers in cord plasma

A total of 570 participants were included in the current study. Acetaminophen, acetaminophen glucuronide and 3-(N-acetyl-L-cysteine-S-yl)-acetaminophen were detected in 16.8%, 17.5% and 28.9% of the cord plasma samples, respectively. Distributions of the raw intensities from liquid chromatography-tandem mass spectrometry (LC-MS) and the corresponding background noise levels are shown in Additional file 1: Fig. 1. We observed high detection agreement among the three acetaminophen biomarkers measured. Among samples with detectable acetaminophen, 92% also had detectable acetaminophen glucuronide and 95% had detectable 3-(N-acetyl-L-cysteine-S-yl)-acetaminophen.

### Characteristics of study participants

Table 1 shows the characteristics of study participants, by detection of acetaminophen. Overall, 66% of the participants had a non-Hispanic black mother, 28% had a Hispanic mother, and 6% had a non-Hispanic white mother. We observed higher proportions of Hispanic mother and non-Hispanic white mother and a lower proportion of non-Hispanic black mother among participants with detectable cord acetaminophen than the not detected group. In addition, participants with detectable acetaminophen in cord plasma had lower gestational age at birth, lower birthweight and were more likely to have primiparous parity, maternal chronic diabetes, maternal preeclampsia and intrauterine inflammation, compared to participants without detectable acetaminophen in cord plasma.

**Table 1:** Maternal and child characteristics, by detection of acetaminophen

Variable	Detection of acetaminophen in cord plasma (*n* = 570)		*P*-value
	No. not detected	No. detected	
Number of participants	474 (83.2%)	96 (16.8%)	
Male	261 (55.1%)	54 (56.2%)	0.92
Maternal race/ethnicity			**0.03**
Non-Hispanic white	27 (5.7%)	9 (9.4%)	
Non-Hispanic black	322 (67.9%)	52 (54.2%)	
Hispanic	125 (26.4%)	35 (36.5%)	
Gestational age at birth in weeks, mean (SD)	38.79 (2.20)	37.75 (3.18)	**< 0.001**
Preterm birth (gestational age < 37 weeks)	69 (14.6%)	31 (32.3%)	**< 0.001**
Birth weight in kilograms, mean (SD)	3.14 (0.64)	2.96 (0.78)	**0.02**
Low birth weight (birth weight < 2500 g)	81 (17.1%)	19 (19.8%)	0.63
Parity: primiparous	199 (42.0%)	53 (55.2%)	**0.02**
Cesarean section	147 (31.0%)	36 (37.5%)	0.26
Intrauterine inflammation	44 (9.3%)	26 (27.1%)	**< 0.001**
Maternal education			0.90
High school or less	312 (65.8%)	65 (67.7%)	
Some college	105 (22.2%)	21 (21.9%)	
College degree or above	57 (12.0%)	10 (10.4%)	
Maternal marital status: not married	310 (65.4%)	69 (71.9%)	0.27
Maternal age in years at delivery, mean (SD)	28.34 (6.56)	27.65 (6.61)	0.35
Any maternal smoking during pregnancy	91 (19.2%)	20 (20.8%)	0.82
Any maternal alcohol use during pregnancy	34 (7.2%)	12 (12.5%)	0.12
Maternal diabetes mellitus			**0.03**
Chronic diabetes	19 (4.0%)	10 (10.4%)	
Gestational diabetes	33 (7.0%)	6 (6.2%)	
No diabetes	422 (89.0%)	80 (83.3%)	
Maternal preeclampsia or HELLP syndrome	29 (6.1%)	22 (22.9%)	**< 0.001**
Maternal pre-pregnancy BMI, mean (SD)	26.65 (6.25)	26.57 (6.11)	0.91
Maternal stress level during pregnancy			0.60
Not stressful	186 (39.2%)	33 (34.4%)	
Average	205 (43.2%)	43 (44.8%)	
Very stressful	83 (17.5%)	20 (20.8%)	

Bold values indicate statistical significance with an alpha level less than 0.05

*SD* standard deviation, *BMI* body mass index, *HELLP* hemolysis, elevated liver enzymes and low platelets. Data are presented as number (percentage) of individuals unless otherwise indicated. *P*-values were obtained using Pearson *χ*^2^ tests for categorical variables and Student’s *t* test for continuous variables

### *CYP2E1* methylation level differs among offspring with detectable acetaminophen

**Fig. 2:**
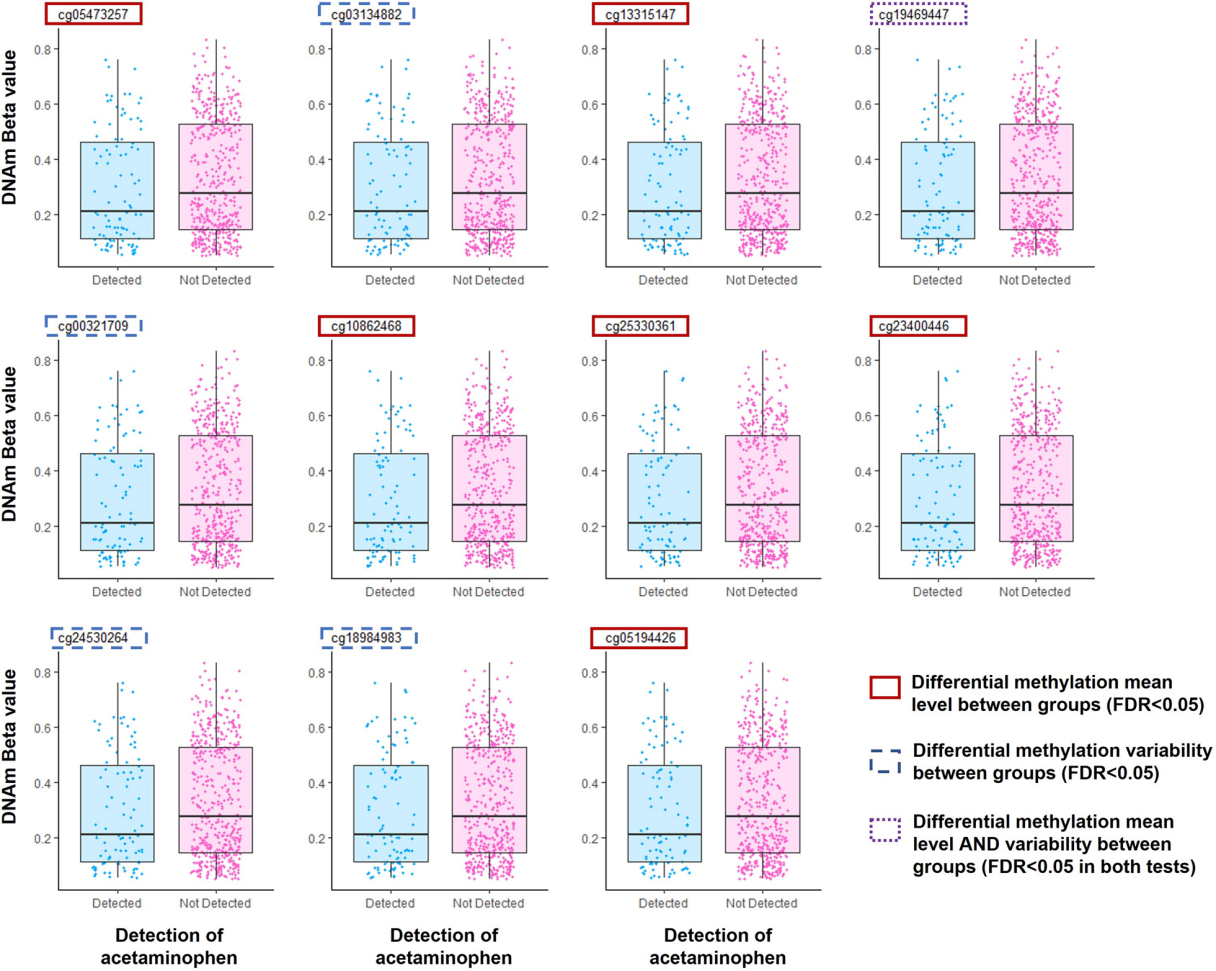
Boxplots for CpG sites showing statistically significant differences in DNA methylation (DNAm) by acetaminophen detection. The y-axis shows DNA methylation levels ranging from 0 to 1 for 0–100% methylated. Blue boxes and dots denote DNAm levels for participants in the detectable acetaminophen group (*n* = 96). Pink boxes and dots denote DNAm levels of participants with no detectable acetaminophen (*n* = 474). The boxplots are arranged from left to right and top to bottom according to their genomic physical position, from upstream (5’) to downstream (3’). Red rectangles mark the differentially methylated CpGs that pass an FDR < 0.05 significance level. Blue rectangles mark the significantly variably methylated CpGs (FDR < 0.05). Purple rectangle marks the CpG with FDR < 0.05 in both the tests

Results of adjusted linear regression models to test for differential methylation between participants with and without detectable acetaminophen level at each of the 28 CpGs tested at the *CYP2E1* gene locus are shown in Table 2. We identified 7 CpGs showing significant differences in DNA methylation levels associated with the detection of acetaminophen in cord blood at an FDR threshold of 0.05. Adjusted linear regression models to test for differential methylation associated with detection of each of the other two acetaminophen metabolites (acetaminophen glucuronide detection and 3-(*N*-acetyl-L-cystein-*S*-yl)-acetaminophen) showed similar results detection (Additional file 1: Tables 1 and 2). In all 7 of the differentially methylated CpGs, participants with detectable acetaminophen had a lower DNA methylation level on average compared to participants without detectable acetaminophen (Fig. 2). The top signal was found at cg13315147 (*p* = 9.84 × 10^−4^, FDR = 0.028) which also reached a conservative Bonferroni significance threshold (*p* = 1.79 × 10^−3^). At this CpG, the mean DNA methylation level was 4.34% (95% CI: 0.8%, 7.9%) lower in the group exposed to acetaminophen. Among the differential methylated CpGs, 6 CpGs were located at CpG island, and 1 CpG was located within 2 kilobases downstream from the CpG island at the shore region (Table 2).

**Table 2:** Results of adjusted linear regression models testing for differential methylation

CpG site	%Difference (95% CI) in DNAm: comparing detected group with not detected group	*P*-value (robust)	FDR	Genomic coordinate (GRCh37/hg19)	Gene Name	Feature category^a^	Relation to CpG Island
cg13315147	− 4.34% (− 7.9%, − 0.8%)	9.84E*−*04*	**0.028**	chr10:135341528	*CYP2E1*	Body	Island
cg05473257	− 4.71% (− 8.6%, − 0.9%)	3.43E*−*03	**0.038**	chr10:135341443	*CYP2E1*	Body	Island
cg23400446	− 4.31% (− 7.9%, − 0.7%)	4.09E*−*03	**0.038**	chr10:135342560	*CYP2E1*	Body	Island
cg05194426	− 5.77% (− 10.6%, − 1%)	9.85E*−*03	**0.045**	chr10:135343193	*CYP2E1*	Body	South Shore
cg19469447	− 3.98% (− 7.2%, − 0.7%)	1.01E*−*02	**0.040**	chr10:135341870	*CYP2E1*	Body	Island
cg10862468	− 3.76% (− 6.9%, − 0.6%)	1.12E*−*02	**0.045**	chr10:135342218	*CYP2E1*	Body	Island
cg25330361	− 3.56% (− 6.2%, − 0.9%)	7.69E*−*03	**0.045**	chr10:135342413	*CYP2E1*	Body	Island
cg03134882	− 3.95% (− 7.3%, − 0.6%)	1.80E*−*02	0.063	chr10:135341463	*CYP2E1*	Body	Island
cg19571004	− 1.26% (− 2.5%, 0%)	2.88E*−*02	0.086	chr10:135340850	*CYP2E1*	TSS200	North Shore
cg00321709	− 5.75% (− 10.2%, − 1.3%)	3.08E*−*02	0.086	chr10:135341933	*CYP2E1*	Body	Island
cg19721068	0.91% (− 0.2%, 2%)	4.60E*−*02	0.117	chr10:135346592	*CYP2E1*	Body	Open sea
cg11445109	− 2.98% (− 6%, 0%)	5.31E*−*02	0.124	chr10:135343248	*CYP2E1*	Body	South Shore
cg18984983	− 3.21% (− 5.8%, − 0.6%)	9.61E*−*02	0.207	chr10:135342936	*CYP2E1*	Body	South Shore
cg24530264	− 3.14% (− 5.6%, − 0.7%)	1.14E*−*01	0.228	chr10:135342620	*CYP2E1*	Body	South Shore
cg00436603	0.62% (− 0.6%, 1.8%)	2.98E*−*01	0.557	chr10:135340740	*CYP2E1*	TSS200	North Shore
cg26065573	0.44% (− 0.2%, 1%)	3.48E*−*01	0.609	chr10:135339469	*CYP2E1*	TSS1500	North Shore
cg09208540	− 0.19% (− 0.6%, 0.3%)	4.07E*−*01	0.671	chr10:135340467	*CYP2E1*	TSS1500	North Shore
cg21024264	− 0.55% (− 1.9%, 0.8%)	4.65E*−*01	0.686	chr10:135341025	*CYP2E1*	1stExon	North Shore
cg07381788	− 0.15% (− 0.8%, 0.5%)	4.60E*−*01	0.686	chr10:135340445	*CYP2E1*	TSS1500	North Shore
cg01355198	− 0.09% (− 0.6%, 0.4%)	5.04E*−*01	0.706	chr10:135347330	*CYP2E1*	Body	Open sea
cg05417377	0.18% (− 0.9%, 1.3%)	6.17E*−*01	0.823	chr10:135350807	*CYP2E1*	Body	Open sea
cg16538390	− 0.15% (− 0.8%, 0.5%)	6.89E*−*01	0.838	chr10:135344917	*CYP2E1*	Body	South Shelf
cg14250048	− 0.02% (− 1%, 1%)	6.86E*−*01	0.838	chr10:135340785	*CYP2E1*	TSS200	North Shore
cg01465364	− 0.06% (− 1.3%, 1.1%)	8.47E*−*01	0.949	chr10:135340721	*CYP2E1*	TSS200	North Shore
cg10986462	0.46% (− 1.4%, 2.3%)	8.34E*−*01	0.949	chr10:135340539	*CYP2E1*	TSS1500	North Shore
cg19837601	0.09% (− 1.3%, 1.5%)	8.91E*−*01	0.960	chr10:135340871	*CYP2E1;CYP2E1*	5'UTR;1stExon	North Shore
cg08472147	0.03% (− 1%, 1%)	9.67E*−*01	0.994	chr10:135340583	*CYP2E1*	TSS1500	North Shore
cg00720244	0.06% (− 0.4%, 0.5%)	9.94E*−*01	0.994	chr10:135347434	*CYP2E1*	Body	Open sea

Results are shown at each of the 28 CpGs located in *CYP2E1*, between participants with (*n* = 96) and without (*n* = 474) acetaminophen detection, sorted by *P*-value

*DNAm* DNA methylation, *FDR* false discovery rate, *chr* chromosome. Difference in percent DNAm was calculated from adjusted linear regression models with DNAm beta value as the dependent variable. *P*-value and FDR were obtained from adjusted linear regression models with DNAm M-value as the dependent variables. The *P*-values are the original *P*-values before adjustment for multiple testing. CpG sites with *P*-value < 1.79 × 10^−3^ (Bonferroni corrected threshold) were marked with asteroid (*). CpG sites with FDR < 0.05 were bolded. Covariates are child sex, delivery type, parity, gestational age, birthweight, maternal age, maternal marital status, prenatal smoking, prenatal alcohol use, intrauterine inflammation, preeclampsia, diabetes mellitus, maternal stress, estimated cell types and 2 surrogate variables. ^a^Gene element type obtained from UCSC database: TSS, transcription start site; TSS200, 200 bases from TSS; TSS1500, 1500 bases from TSS; UTR, untranslated region

As a sensitivity analysis, we further adjusted for maternal race and ethnicity in the linear regression models to evaluate whether this variable may explain the associations found in the main analysis. The results did not change after the further adjustment, suggesting that the association between cord acetaminophen and methylation at *CYP2E1* was unlikely driven by maternal race or ethnicity (Additional file 1: Table 3).

A previous study reported that maternal gestational diabetes was associated with lower cord blood methylation level at the *CYP2E1* [11]. Although maternal diabetes status (chronic vs. gestational vs. no diabetes) has been adjusted as a covariate in the main analysis to account for the potential confounding effect, we performed additional sensitivity analysis to make sure our results were not driven by data from a small proportion of participants with maternal gestational diabetes. In the subgroup of participants without maternal gestational diabetes exposure (*n* = 531), methylation levels at the 7 differentially methylated CpGs were still significantly lower in participants with detectable acetaminophen than those without, and the effect sizes remained similar (Additional file 1: Table 4). These results suggested that the association between cord acetaminophen and methylation at *CYP2E1* was unlikely to be driven by gestational diabetes.

### Methylation variation at *CYP2E1* and acetaminophen detection

Results from adjusted statistical models to test for differences in methylation variation related to acetaminophen detection at each of the 28 CpGs in *CYP2E1* are shown in Table 3. We identified 5 CpGs showing different variability in methylation levels associated with the detection of acetaminophen in cord blood at an FDR threshold of 0.05. Among them, 4 CpGs also passed a conservative Bonferroni significance threshold (*p* < 1.79 × 10^−3^). At all 5 CpGs, the variance in methylation levels was smaller among participants with detectable cord acetaminophen than those without. The top signal was found at cg24530264 (*p* = 2.70 × 10^−4^, FDR = 0.008) where the variance of methylation M-value in the detected group was 0.73 times the variance in the not detected group (log variance ratio = − 0.303). Among the variably methylated CpGs, 3 were located at a CpG island and 2 were located within 2 kilobases downstream from the CpG island (Table 3). As shown in Fig. 2, the average DNA methylation level at the 5 variably methylated CpGs was also lower among participants with detectable acetaminophen compared to participants without detectable acetaminophen. Similar results were observed for the other 2 acetaminophen metabolites measured (Additional file 1: Tables 5 and 6).

**Table 3:** Results of adjusted models testing for differences in methylation variation

CpG site	Sample variance	Log variance ratio	Difference in Levene’s Residuals	*P*-value (robust)	FDR	Genomic coordinate (GRCh37/hg19)	Gene Name	Feature category^a^	Relation to CpG Island
cg24530264	0.888	− 0.303	− 0.179	2.70E*−*04*	**0.008**	chr10:135342620	*CYP2E1*	Body	South Shore
cg18984983	1.004	− 0.364	− 0.168	9.94E*−*04*	**0.012**	chr10:135342936	*CYP2E1*	Body	South Shore
cg00321709	1.115	− 0.260	− 0.180	1.58E*−*03*	**0.012**	chr10:135341933	*CYP2E1*	Body	Island
cg03134882	0.805	− 0.145	− 0.126	1.68E*−*03*	**0.012**	chr10:135341463	*CYP2E1*	Body	Island
cg19469447	1.296	− 0.142	− 0.140	4.07E*−*03	**0.023**	chr10:135341870	*CYP2E1*	Body	Island
cg13315147	0.818	− 0.119	− 0.097	1.70E*−*02	0.079	chr10:135341528	*CYP2E1*	Body	Island
cg05473257	1.327	− 0.116	− 0.108	2.69E*−*02	0.108	chr10:135341443	*CYP2E1*	Body	Island
cg25330361	0.516	− 0.214	− 0.095	3.75E*−*02	0.131	chr10:135342413	*CYP2E1*	Body	Island
cg23400446	1.423	− 0.090	− 0.090	1.00E*−*01	0.312	chr10:135342560	*CYP2E1*	Body	Island
cg21024264	0.139	0.245	0.041	1.42E*−*01	0.398	chr10:135341025	*CYP2E1*	1stExon	North Shore
cg26065573	0.081	− 0.241	− 0.028	1.64E*−*01	0.410	chr10:135339469	*CYP2E1*	TSS1500	North Shore
cg09208540	0.034	0.162	0.019	1.76E*−*01	0.410	chr10:135340467	*CYP2E1*	TSS1500	North Shore
cg07381788	0.063	− 0.121	− 0.021	2.58E*−*01	0.555	chr10:135340445	*CYP2E1*	TSS1500	North Shore
cg11445109	1.201	− 0.144	− 0.074	3.12E*−*01	0.595	chr10:135343248	*CYP2E1*	Body	South Shore
cg01355198	0.065	− 0.120	− 0.019	3.19E*−*01	0.595	chr10:135347330	*CYP2E1*	Body	Open Sea
cg19837601	0.104	0.031	0.022	3.91E*−*01	0.685	chr10:135340871	*CYP2E1;CYP2E1*	5'UTR;1stExon	North Shore
cg10862468	0.578	0.016	− 0.035	4.17E*−*01	0.687	chr10:135342218	*CYP2E1*	Body	Island
cg05417377	0.074	0.100	0.015	4.42E*−*01	0.687	chr10:135350807	*CYP2E1*	Body	Open Sea
cg08472147	0.046	− 0.070	− 0.011	5.04E*−*01	0.719	chr10:135340583	*CYP2E1*	TSS1500	North Shore
cg10986462	0.241	0.068	− 0.031	5.14E*−*01	0.719	chr10:135340539	*CYP2E1*	TSS1500	North Shore
cg00720244	0.063	− 0.114	− 0.010	5.93E*−*01	0.790	chr10:135347434	*CYP2E1*	Body	Open Sea
cg19721068	0.107	− 0.440	− 0.011	6.81E*−*01	0.867	chr10:135346592	*CYP2E1*	Body	Open Sea
cg14250048	0.115	− 0.225	0.007	7.84E*−*01	0.954	chr10:135340785	*CYP2E1*	TSS200	North Shore
cg00436603	0.104	− 0.019	− 0.003	9.04E*−*01	0.973	chr10:135340740	*CYP2E1*	TSS200	North Shore
cg16538390	0.038	0.098	− 0.001	9.35E*−*01	0.973	chr10:135344917	*CYP2E1*	Body	South Shelf
cg19571004	0.162	0.076	− 0.002	9.46E*−*01	0.973	chr10:135340850	*CYP2E1*	TSS200	North Shore
cg05194426	1.161	0.023	0.002	9.72E*−*01	0.973	chr10:135343193	*CYP2E1*	Body	South Shore
cg01465364	0.113	− 0.006	0.001	9.73E*−*01	0.973	chr10:135340721	*CYP2E1*	TSS200	North Shore

Results are shown at each of the 28 CpGs located in *CYP2E1*, between participants with (*n* = 96) and without (*n* = 474) acetaminophen detection, sorted by *P*-value

*DNAm* DNA methylation, *FDR* false discovery rate, *chr* chromosome. The *P*-values are the original *P*-values before adjustment for multiple testing. CpG sites with *P*-value < 1.79 × 10^−3^ (Bonferroni corrected threshold) were marked with asteroid (*). CpG sites with FDR < 0.05 were bolded. Covariates are child sex, delivery type, parity, gestational age, birthweight, maternal age, maternal marital status, prenatal smoking, prenatal alcohol use, intrauterine inflammation, preeclampsia, diabetes mellitus, maternal stress, estimated cell types and 2 surrogate variables. ^a^Gene feature category of CpG sites obtained from UCSC database: TSS, transcription start site; TSS200, 200 bases from TSS; TSS1500, 1500 bases from TSS; UTR, untranslated region

Further adjustment for maternal race and ethnicity in the statistical models did not change the results (Additional file 1: Table 7). Sensitivity analyses among the subgroup of participants without maternal gestational diabetes (*n* = 531) remained the same (Additional file 1: Table 8). These results suggested that our findings of variable methylation at *CYP2E1* associated with the detection of acetaminophen were not driven by maternal race, ethnicity or gestational diabetes.

**Fig. 3:**
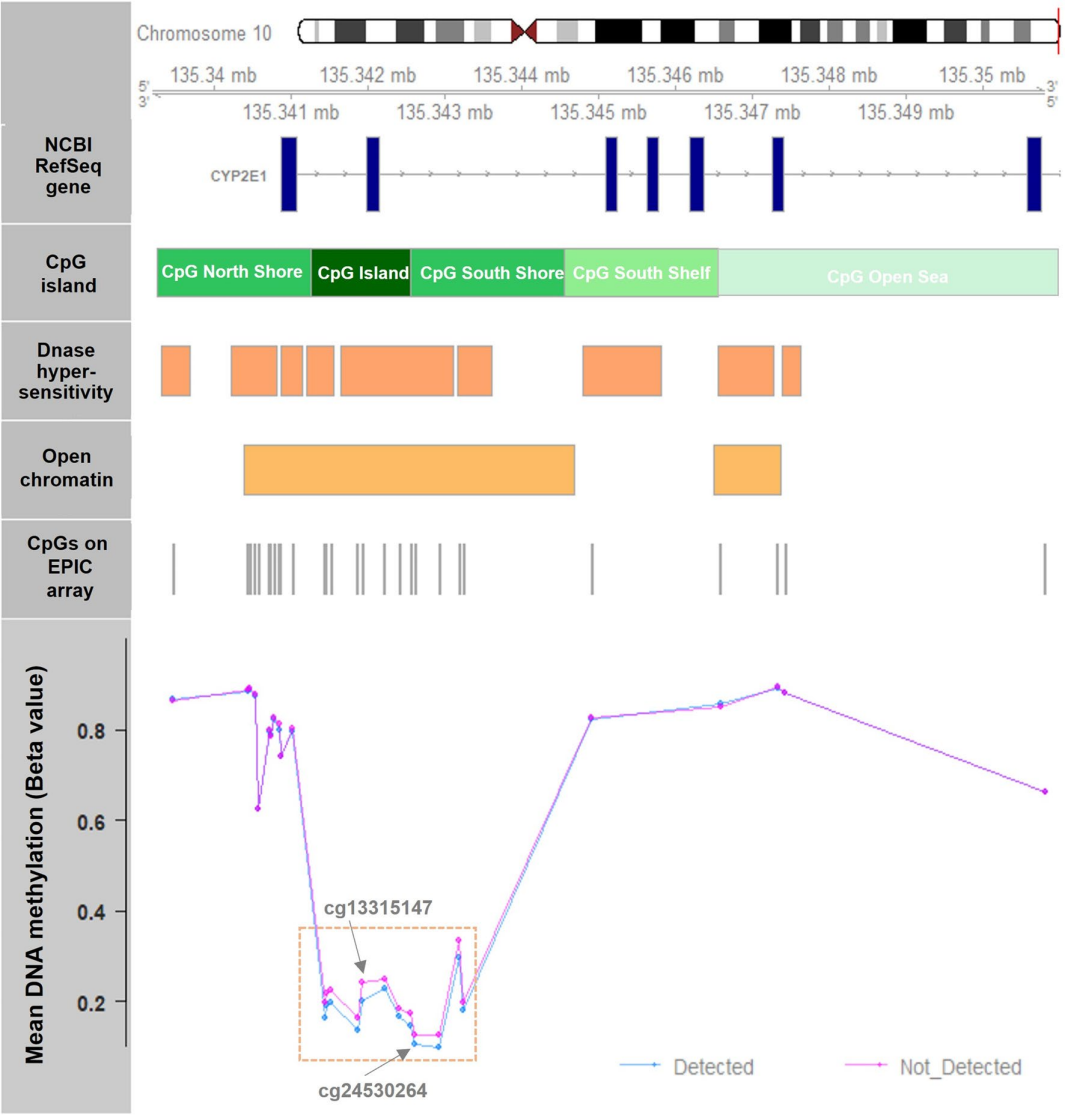
Gene annotations and DNA methylation levels at the *CYP2E1* candidate genomic region. The 28 CpG sites tested in the *CYP2E1* gene located on chromosome 10 at position 135339000–135351000 (GRCh37/hg19; shown in top panel). Gene annotation categories are provided on the left side in the grey panel, with exons in blue, CpG island relationships in green rectangles, ENCODE DNase hypersensitive sites are shown in dark orange and open chromatin in light orange. Grey tick marks indicate the location of CpG sites measured on the Illumina EPIC array. Annotations were derived from the NCBI Reference Sequence (RefSeq) Database. The bottom panel shows the mean percent methylation detected at each CpG site for samples with detectable levels of acetaminophen metabolites (pink) and those with undetectable levels (blue). The dashed gold rectangle highlights the region where methylation showed either significant mean shift or different variation related to acetaminophen detection

Figure 3 visualizes the gene annotations and DNA methylation levels at *CYP2E1* genomic region. The CpGs where DNA methylation showed significant mean shift or different variation are located at a known CpG island in the UCSC database (chr10: 135341256–135342561, GRCh37/hg19) or within 1 kilobase downstream from the island. The mean DNA methylation levels at these sites (mean beta-value: 0.15–0.35) were lower than the levels at surrounding CpG sites (mean beta value: 0.60–0.90). The CpG sites showed differential or variably methylation span from the 1^st^ intron to the 2nd intron of *CYP2E1* and overlap with DNase hypersensitive regions and an open chromatin region identified by experimental data from the ENCODE project [12].

## Discussion

In this sample of 570 children representing an urban, low-income, multi-racial ethnic population from Boston, we found that perinatal acetaminophen exposure, detected via cord plasma metabolites, was associated with lower DNA methylation levels at several CpG sites located in the 5’ region of *CYP2E1*. Many of these CpG sites locate within the first intron region of *CYP2E1* where demethylation is known to be associated with higher *CYP2E1* transcription in fetal liver and placenta [8–10]. In addition, we observed less variation in methylation levels at multiple CpG sites of *CYP2E1* among neonates with detectable levels of acetaminophen at birth compared to those without. The CpG sites we identified are in close proximity to one another and overlap with known DNase hypersensitivity and open chromatin regions. This evidence further supported that the CpG sites are likely to have important roles in the epigenetic regulation and transcriptional activation of *CYP2E1*.

Notably, at all the CpG sites showing a significant difference in methylation variation, we also observed a lower average methylation level among neonates with an acetaminophen detection than neonates without. The %difference in methylation at these sites was comparable to those at differentially methylated CpG sites (Fig. 2). Linear regression assumes equal variance (i.e., the variance of dependent variable is constant at any level of an independent variable). However, at the CpGs we identified as variably methylated, the assumption of equal variance was not met. To address this concern, we performed an ad hoc analysis using weighted linear regression to test for differential methylation. The results remain largely unchanged.

Moreover, we observed a larger sample variance of methylation at the differential and/or variably methylated CpGs (located at CpG island or South Shore) compared to the surrounding CpGs (Table 3), suggesting that DNA methylation at these sites is likely under the influence of some upstream factors, either environmental or genetic, and their interactions. A previous study found that genetic polymorphism can affect the methylation level of *CYP2E1* in newborns [13]. It is possible that the association between acetaminophen and *CYP2E1* DNA methylation varies by genotype, particularly at CpG sites with different DNA methylation variance. Unfortunately, we were unable to directly assess this aspect as genetic data are not currently available for this analysis, which we recognize as a limitation. Further studies should incorporate genetic data to gain further insight into the complex interplay between genetics, acetaminophen exposure and DNA methylation in relation to *CYP2E1* regulation.

Acetaminophen has a relatively short half-life, about 3–4 h, in pregnant women [14]. In this study, we measured acetaminophen exposure in cord plasma, which captured maternal acetaminophen use shortly before delivery. The expression of CYP2E1 protein in the human fetal liver starts as early as in the second trimester, and the expression level increases in the third trimester and perinatal period [15]. Before the end of the second trimester, fetal liver has no or little expression of CYP2E1 as a result of DNA methylation at the 5’ end of *CYP2E1* [8, 15]. Therefore, we postulate that late pregnancy to delivery might be the critical time window for in-utero acetaminophen exposure to influence fetus through CYP2E1 expression. Determining the relevant exposure window of prenatal acetaminophen, if it exists, will be highly valuable for improving current clinical recommendations.

Previous EWAS has scanned the genome to identify CpG sites showing differential methylation in cord blood [16, 17] and placenta [18] related to prenatal exposure to acetaminophen. However, they did not identify CpG sites in *CYP2E1*, after correcting for multiple comparisons. Several major differences between our study and the previous EWAS, including study design, characteristics of participants, exposure measurement method and timing, and biospecimen type could potentially explain the inconsistency. Additionally, previous EWAS studies are likely still underpowered to detect the methylation changes at *CYP2E1* associated with acetaminophen exposure. In fact, at all the CpG sites of *CYP2E1* we identified in the study, the directions of effect are consistent with the results from the placenta EWAS [18] in extremely low gestational age newborns.

In a review article [19], it is highlighted that various prenatal exposures are associated with small-magnitude DNA methylation changes, often below 5%. Importantly, these changes have been consistently replicated across different populations and over time. In the current study, we observed DNA methylation differences between the groups with detectable and undetectable acetaminophen levels at multiple CpG sites, with effect sizes ranging from 3 to 6%. Significantly, these CpG sites are physically adjacent to each other and overlap with regions of DNase hypersensitivity and open chromatin in the 5’ region of *CYP2E1* (Fig. 3), which are known to be involved in the transcriptional regulation of the gene [8–10]. Therefore, despite their small effect sizes, we believe that these observed associations are likely to have downstream biological impacts. It is important to note that in our study, DNA methylation was measured in cord blood, which represents a bulk tissue comprising multiple cell types. It is possible that one or a few cell types within cord blood undergo a large methylation shift at those CpGs; however, the large effects were diluted on the tissue level due to the small proportions of the relevant cell types. To gain further insights and identify the relevant cell types, studies incorporating improved cell-type resolution in DNA methylation measurements are warranted.

A major strength of our study is the use of rich empiric molecular data obtained from BBC participants, which allowed us to evaluate the association between an objective measure of acetaminophen exposure, i.e., cord plasma metabolite measures, and *CYP2E1* cord blood DNA methylation measures. The objectively measured acetaminophen biomarkers were less prone to measurement errors due to inaccurate self-report and were able to capture acetaminophen exposure during a specific short time window before delivery.

We observed significant differences in maternal pregnancy conditions and birth outcomes between individuals with detectable and undetectable acetaminophen metabolites in cord blood. Specifically, mothers of neonates with detectable acetaminophen showed a higher prevalence of intrauterine inflammation, characterized by common symptoms such as intrapartum maternal fever [20], and preeclampsia, indicated by symptoms like severe headache and epigastric pain [21]. These pregnancy conditions are known risk factors for preterm birth and low birth weight [22, 23], which may explain the significant differences in these birth outcomes between the acetaminophen groups. These observations underscore the importance of carefully considering potential confounding by indication in our analysis. To address this concern, we adjusted for relevant maternal clinical variables during pregnancy, aiming to minimize the potential impact of confounding. Furthermore, we conducted sensitivity analysis and observed similar changes in methylation among newborns whose mother did not have gestational diabetes. This suggested that the results were not driven by residual confounding due to gestational diabetes, a pregnancy condition that has been previously associated with *CYP2E1* DNA methylation [11]. However, it is important to acknowledge that despite our best efforts, unmeasured confounding remains a potential limitation due to the inherent nature of observational data in this study.

Existing literature has shown that decrease in DNA methylation at the 5’ region of *CYP2E1* is known to result in an increased gene expression in fetal liver [9], possibly through modulating the hepatocyte nuclear factor 1 alpha (HNF-1α) transcription factor binding [24, 25]. This upregulation in *CYP2E1* gene expression can lead to elevated production of NAPQI, the toxic metabolite of acetaminophen [5, 6], which may have implications for fetal development. We acknowledge that the current study was limited by the unavailability of fetal liver tissue samples, which would have provided direct insight into the biological mechanisms regarding acetaminophen metabolism. However, it is important to note that collecting fetal liver tissue requires invasive procedures, making it impractical for large-scale population-based studies.

**Fig. 4:**
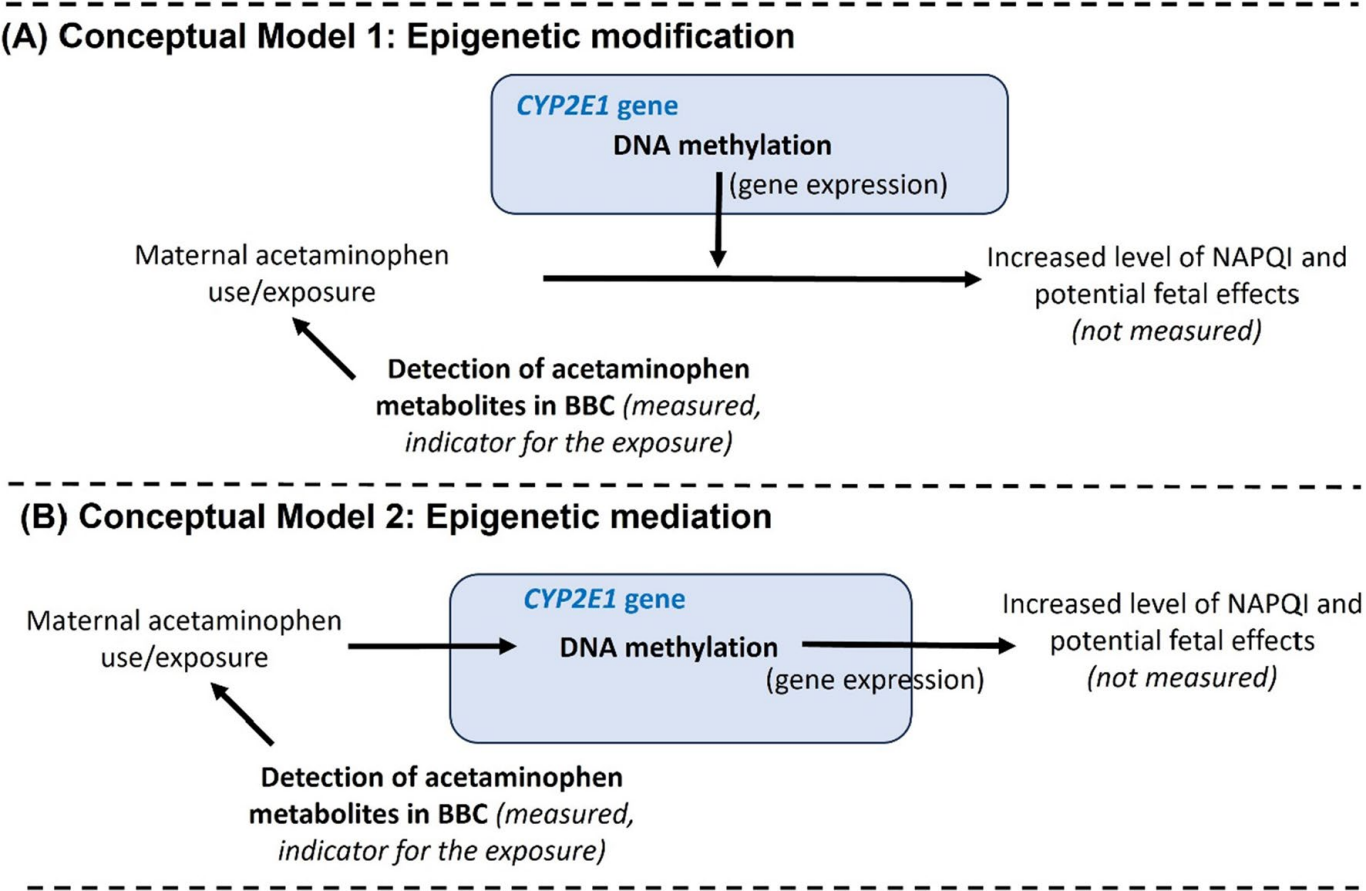
Potential conceptual models underlying the observed association between acetaminophen metabolites and DNA methylation at *CYP2E1* in the Boston Birth Cohort (BBC)

In this study, we considered the measured acetaminophen metabolites as indicators of maternal acetaminophen exposure, because unlike NAPQI (the toxic metabolite), production of these metabolites is not directly related to CYP2E1 enzyme activities. We propose two conceptual models that could explain the observed association between acetaminophen metabolites and DNA methylation at the *CYP2E1* locus (Fig. 4). DNA methylation of *CYP2E1* may modify the effect of maternal acetaminophen exposure on production of NAPQI and its potential fetal effects (Fig. 4A). It is also possible that DNA methylation of *CYP2E1* lies on the causal pathway from maternal acetaminophen exposure to NAPQI production and acts as a mediator (Fig. 4B). However, due to the study design and the absence of NAPQI measurements in the current study, we were unable to differentiate between these two models. Nevertheless, the findings from this study are significant as they provide crucial initial evidence in population-based cohort, demonstrating an association between acetaminophen metabolites and *CYP2E1* DNA methylation level. This association underscores the need for further investigation into the specific biological mechanisms involved, whether they pertain to effect modification or mediation by DNA methylation at the *CYP2E1* locus. Future studies employing longitudinal data and/or experimental designs, along with the inclusion of direct measurement of acetaminophen toxicity, are warranted to elucidate the effects of maternal acetaminophen exposure on fetal development and the precise roles of DNA methylation in regulating this process.

Our study population mainly consisted of urban, low-income, racially and ethnically diverse individuals from Boston, Massachusetts. In order to assess the generalizability of our findings, it is essential that future studies investigate the associations between maternal acetaminophen exposure before delivery and newborn’s *CYP2E1* methylation in populations with different characteristics and demographics.

## Conclusions

In this US birth cohort, for the first time, we examined the association between objective measurements of acetaminophen metabolites and DNA methylation of the *CYP2E1* gene in cord blood. Our analyses revealed a notably association between the presence of acetaminophen metabolites and a reduced level of DNA methylation in the 5’ region of *CYP2E1*, a gene known to play important roles in regulating acetaminophen toxicity. The findings provide critical initial evidence in newborns, indicating that maternal acetaminophen exposure during late pregnancy may influence fetal development. Future studies are warranted to replicate our results in other populations and to further investigate whether the *CYP2E1* DNA methylation associated with maternal acetaminophen exposure may have implications for child’s short- and long-term health outcomes.

## Methods

### Study design and population

The Boston Birth Cohort (BBC) is a longstanding, prospective cohort study initiated in 1998 that enrolls mothers who deliver at the Boston Medical Center (BMC) and their child. A detailed study protocol has been published [26]. Briefly, BBC represents an urban, low-income and multi-ethnic population in the Boston area, Massachusetts, USA. Umbilical cord blood samples were collected at delivery and were separated into plasma, white blood cells and red blood cells and stored in freezer at − 80 °C. After enrollment at delivery, a subset of children who continued to receive pediatric care at the BMC were prospectively followed up until 21 years of age. For the current analysis, we included 570 BBC children who have available data on cord blood DNA methylation and acetaminophen metabolites measured in cord plasma. The included participants were enrolled between December 2003 and October 2013.

The BBC study protocol was approved by the Institutional Review Boards of the Boston Medical Center and the Johns Hopkins Bloomberg School of Public Health. Written consent was obtained from all participating mothers.

### Cord blood sample acquisition and processing

Umbilical cord blood samples were collected by trained nursing staff at delivery through venous umbilical cord milking using a BD Vacutainer® Plus Plastic K20EDTA tube. The samples were centrifuged (0° Celsius at 1430 g for 13 min) and fractionated into plasma, white blood cells and red blood cells and were stored in a freezer at − 80 °C.

### Measurement of acetaminophen biomarkers in cord plasma

Quantitative profiling of metabolites, including acetaminophen, acetaminophen glucuronide and 3-(*N*-acetyl-L-cystein-*S*-yl)-acetaminophen, was conducted in the umbilical cord plasma samples using liquid chromatography-tandem mass spectrometry (LC-MS) following established protocol [27] at the Harvard-MIT Broad Institute Metabolite Profiling Laboratory. We applied multiple quality control and quality assurance procedures. Briefly, a pooled reference sample composed of all individual study samples was randomly inserted across samples (per 20–30 samples) and the coefficient of variation (CV) was calculated for each metabolite using the reference samples. The CV for acetaminophen, acetaminophen glucuronide and 3-(*N*-acetyl-L-cystein-*S*-yl)-acetaminophen was 0.07, 0.06 and 0.03, respectively, suggesting high reliability of the measurements. We closely inspected the raw intensity output from the LC-MS and determined the background noise levels for the acetaminophen metabolites. We created a dichotomized variable (detected versus not detected) based on the background noise level for each acetaminophen metabolite.

### Measurement of DNA methylation and quality controls

Genomic DNA was isolated from EDTA-treated white blood cells and diluted to 50–100 ng/uL. The diluted samples were sent to the University of Minnesota Genomics Center for genome-wide DNA methylation profiling using the Illumina Infinium MethylationEPIC BeadChip platform. With the EPIC platform, DNA methylation levels for a total of 865,859 CpG sites were generated, offering extensive coverage of CpG islands, genes and enhancers across the genome. Sample-level and probe-level quality control procedures were conducted following the existing pipeline implemented by the R/Bioconductor package “minfi” [28]. Specifically, samples with low overall intensity (median log2 intensity less than 10) or probe missing rates exceeding 2% were excluded from the analysis. Outliers were identified and removed based on multi-dimensional scaling (MDS) plots. The biological sex of each sample was estimated using DNA methylation data, and any samples with inconsistencies between the DNA methylation-derived sex and the parent-reported sex in the medical records were excluded from subsequent analysis. Probes were also eliminated if they met any of the following criteria: 1) failed detection (detP > 0.01) in over 5% of the samples, 2) overlapped with a SNP at the measured or extension site or 3) cross-hybridized to other genomic locations. After the quality control check, we applied the single-sample single-sample normal-exponential out-of-band (ssNoob) methods [29] (*preprocessnoob()* in “minfi”) to correct for background and dye bias and performed quartile normalization (*preprocessQuantile()* in “minfi”) to normalize type I and type II probes.

### DNA methylation level of CYP2E1

We used the Illumina MethylationEPIC v1.0 B5 Manifest file to identify CpG sites for the *CYP2E1* gene. The manifest file can be downloaded from the Illumina website using the link: https://support.illumina.com/array/array_kits/infinium-methylationepic-beadchip-kit/downloads.html. Specifically, we identified 28 CpG sites that are annotated to the *CYP2E1* gene based on the UCSC Genes track (i.e., the “UCSC_RefGene_Name” column in the manifest file contains “*CYP2E1*”). The 28 CpG sites are located on chromosome 10 and mainly cover the 5’ end region (TSS1500, TSS200, 5’UTR, 1st and 2nd exons, 1st and 2nd introns) of *CYP2E1.* Among the 28 candidate CpG sites, 8 of them fall within the region of a CpG island (chr10: 135341256–135342561, GRCh37/hg19) in the UCSC database.

### Estimation of cord blood sample cell composition

We inferred the composition of different cell types (CD4 + T cells, CD8 + T cells, B cells, monocytes, granulocytes, natural killer cells and nucleated red blood cells) in the cord blood samples using the genome-wide DNA methylation data. This calculation was conducted using the *estimateCellCounts()* function in the “minfi” package, which implements a reference-based method developed by Bakulski et al. [30]. We adjusted for the estimated cell-type compositions as covariates in the downstream statistical analysis.

### Surrogate variables to correct for batch effects

To account for potential batch effects*,* we performed surrogate variable analysis (SVA) based on the DNA methylation levels at all CpG sites located on chromosome 10 using the R/Bioconductor package “sva” [31]. All covariates and cell types were included in the SVA model to estimate the surrogate variables. These surrogate variables were included as covariates in the downstream statistical analysis.

### Child and maternal characteristics as covariates

Child and maternal characteristics were collected through maternal interviews at the time of study enrollment within 24–72 h of delivery and electronic medical records (EMR) abstraction. Child covariates were sex, delivery type (cesarean section or vaginal delivery), primiparous parity, gestational age at birth (weeks) and birth weight (grams). Maternal covariates were age at delivery, marital status, any cigarette smoking during pregnancy, any alcohol use during pregnancy, intrauterine inflammation (any placenta histopathology consistent with uterine inflammation that was determined by pathologists or the presence of intrapartum maternal fever > 38 °C at parturition), preeclampsia or HELLP (Hemolysis Elevated Liver enzymes and Low Platelets) syndrome, diabetes mellitus (chronic, gestational or no diabetes) and stress level during pregnancy. In addition, we also collected data on the following maternal variables: race/ethnicity, education and pre-pregnancy body mass index (BMI).

### Statistical analysis

The maternal and child characteristics were compared between the two groups of acetaminophen detection (i.e., detected versus not detected) using Pearson χ^2^ for categorical variables and Student’s t test for continuous variables.

To test whether the mean DNA methylation level at *CYP2E1* was different between the two groups of acetaminophen detection, we fit linear regression models using R/Bioconductor package “limma” [32] for each of the 28 candidate CpG sites. In the linear regression models, DNA methylation (beta-value or M-value) at a specific CpG site was the dependent variable and detection of acetaminophen (dichotomized) was an independent variable. Following recommendations from previous literature [33], we used *p*-values from the models with M-value to assess the statistical significance and reported effect size estimates from the models with beta-value for better interpretations. The following covariates were adjusted in the model: child sex, delivery type, parity, gestational age, birth weight, maternal age, maternal marital status, prenatal smoking, prenatal alcohol use, intrauterine inflammation, preeclampsia, diabetes mellitus, maternal stress, estimated cell-type proportions (CD4 + T cells, CD8 + T cells, B cells, monocytes, granulocytes, natural killer cells and nucleated red blood cells) and surrogate variables. We applied the empirical Bayes approach (*eBaye()* in “limma”) to obtain robust *p*-values for statistical inference. The same linear regression models were fit for acetaminophen glucuronide and 3-(*N*-acetyl-L-cystein-*S*-yl) acetaminophen.

To test whether the variability of DNA methylation at *CYP2E1* was different between the two groups of acetaminophen detection, we applied the DiffVar method [34]. Briefly, DiffVar builds on Levene’s test for equality of variances and employs an empirical Bayes modeling framework to stabilize the test statistics. This method can take into account potential confounders and experimental designs as covariates and is robust to outliers [34]. Here, we used DiffVar to test differential variability of DNA methylation (*M*-value) between the detection groups of acetaminophen biomarkers, respectively, adjusting for the same covariates mentioned above.

We conducted the following sensitivity analyses. First, we further adjusted for maternal race/ethnicity in the linear regression and the DiffVar models to examine whether the findings from the main analysis may be confounded by this variable. Second, we excluded participants with gestational diabetes from the study samples and fit the linear regression and DiffVar models in the rest of the samples.

We reported the *p*-value from the statistical models and the Benjamini–Hochberg false discovery rate (FDR) [35]. CpG sites with FDR less than 0.05 in the linear regression models were determined as differentially methylated positions, and CpG sites with FDR less than 0.05 in the DiffVar tests were determined as variably methylated positions. We also applied the Bonferroni correction to the *p*-value as this method is considered more conservative. The Bonferroni significance threshold was 1.79 × 10^−3^ (i.e., 0.05/28).

We obtained gene annotations for the 28 candidate CpG sites from publicly available databases. Specifically, the chromosomal coordinates of exons and introns of *CYP2E1* were obtained from NCBI Reference Sequence (RefSeq) database [36]. The chromosomal coordinates of the CpG island in the candidate region were obtained from UCSC Genome Brower [37]. We defined the following categories to describe the feature of CpG sites based on their relation to CpG island: North Shore (0–2 kbs upstream from the island), South Shore (0–2 kbs downstream from the island), South Shelf (2–4 kbs downstream from the island) and Open Sea (> 4 kbs from the island). The chromosomal coordinates of DNase hypersensitive regions and open chromatin regions were obtained from the ENCODE project [12] and included in the Illumina Manifest file. We used R/Bioconductor package “Gviz” [38] to visualize the gene annotations for the candidate region.

All statistical analyses were performed using R version 4.1.1 (R Foundation for Statistical Computing).

### Supplementary Information

**Additional file 1:** Additional Tables 1–8.

## Data Availability

The datasets supporting these findings are not publicly available. Instead, the datasets used and/or analyzed for the current study are available from the corresponding author on reasonable request and after institutional review board review and approval.

## Abbreviations

BBC: Boston Birth Cohort
BMC: Boston Medical Center
CpG: 5’-Cytosine-phosphate-guanine-3’
CYP2E1: Cytochrome p450 family 2 subfamily e member 1
CV: Coefficient of variation
DNA: Deoxyribonucleic acid
DNAm: DNA methylation
DNMT3A: DNA methyltransferase 3 alpha
DNMT3B: DNA methyltransferase 3 beta
ENCODE: Encyclopedia of DNA elements
EWAS: Epigenome-wide association study
FDR: False discovery rate
GSH: Glutathione
HELLP: Hemolysis, elevated liver enzymes and low platelets
SD: Standard deviation
LC-MS: Liquid chromatography-tandem mass spectrometry
NAPQI: *N*-acetyl-*p*-benzoquinone imine
SVA: Surrogate variable analysis
TET1: Ten-eleven translocation methylcytosine dioxygenase 1

## References

[1] Werler MM, Mitchell AA, Hernandez-Diaz S, Honein MA (2005). Use of over-the-counter medications during pregnancy. Am J Obstet Gynecol.

[2] Levy G, Garrettson LK, Soda DM (1975). Evidence of placental transfer of acetaminophen. Pediatrics.

[3] Roberts I, Robinson M, Mughal M, Ratcliffe J, Prescott L (1984). Paracetamol metabolites in the neonate following maternal overdose. Br J Clin Pharmacol.

[4] Nitsche JF, Patil AS, Langman LJ (2017). Transplacental passage of acetaminophen in term pregnancy. Am J Perinatol.

[5] Hodgman MJ, Garrard AR (2012). A review of acetaminophen poisoning. Crit Care Clin.

[6] Yoon E, Babar A, Choudhary M, Kutner M, Pyrsopoulos N (2016). Acetaminophen-induced hepatotoxicity: a comprehensive update. J Clin Transl Hepatol.

[7] Lee SST, Buters JTM, Pineau T, Fernandez-Salguero P, Gonzalez FJ (1996). Role of CYP2E1 in the hepatotoxicity of acetaminophen. J Biol Chem.

[8] Vieira I, Sonnier M, Cresteil T (1996). Developmental expression of CYP2E1 in the human liver. Eur J Biochem.

[9] Jones SM, Boobis AR, Moore GE, Stanier PM (1992). Expression of CYP2E1 during human fetal development: methylation of the CYP2E1 gene in human fetal and adult liver samples. Biochem Pharmacol.

[10] Vieira I, Pasanen M, Raunio H, Cresteil T (1998). Expression of CYP2E1 in human lung and kidney during development and in full-term placenta: a differential methylation of the gene is involved in the regulation process. Pharmacol Toxicol.

[11] Howe CG, Cox B, Fore R (2020). Maternal gestational diabetes mellitus and newborn DNA methylation: findings from the pregnancy and childhood epigenetics consortium. Diabetes Care.

[12] Rosenbloom KR, Sloan CA, Malladi VS (2013). ENCODE data in the UCSC genome browser: year 5 update. Nucleic Acids Res.

[13] Zhu Y, Mordaunt CE, Yasui DH (2019). Placental DNA methylation levels at CYP2E1 and IRS2 are associated with child outcome in a prospective autism study. Hum Mol Genet.

[14] Rayburn W, Shukla U, Stetson P, Piehl E (1986). Acetaminophen pharmacokinetics: comparison between pregnant and nonpregnant women. Am J Obstet Gynecol.

[15] Johnsrud EK, Koukouritaki SB, Divakaran K, Brunengraber LL, Hines RN, McCarver DG (2003). Human hepatic CYP2E1 expression during development. J Pharmacol Exp Ther.

[16] Eslamimehr S, Jones AD, Anthony TM (2022). Association of prenatal acetaminophen use and acetaminophen metabolites with DNA methylation of newborns: analysis of two consecutive generations of the Isle of Wight birth cohort. Environ Epigenet.

[17] Gervin K, Nordeng H, Ystrom E, Reichborn-Kjennerud T, Lyle R (2017). Long-term prenatal exposure to paracetamol is associated with DNA methylation differences in children diagnosed with ADHD. Clin Epigenet.

[18] Addo KA, Bulka C, Dhingra R (2019). Acetaminophen use during pregnancy and DNA methylation in the placenta of the extremely low gestational age newborn (ELGAN) cohort. Environ Epigenet.

[19] Breton CV, Marsit CJ, Faustman E (2017). Small-magnitude effect sizes in epigenetic end points are important in children’s environmental health studies: the children’s environmental health and disease prevention research center’s epigenetics working group. Environ Health Perspect.

[20] Hagberg H, Wennerholm UB, Sävman K (2002). Sequelae of chorioamnionitis. Curr Opin Infect Dis.

[21] Thangaratinam S, Gallos ID, Meah N, Usman S, Ismail KMK, Khan KS (2011). How accurate are maternal symptoms in predicting impending complications in women with preeclampsia? A systematic review and meta-analysis. Acta Obstet Gynecol Scand.

[22] Davies EL, Bell JS, Bhattacharya S (2016). Preeclampsia and preterm delivery: a population-based case–control study. Hypertens Pregnancy.

[23] Vrachnis N, Vitoratos N, Iliodromiti Z, Sifakis S, Deligeoroglou E, Creatsas G (2010). Intrauterine inflammation and preterm delivery. Ann N Y Acad Sci.

[24] Liu SY, Gonzalez FJ (1995). Role of the liver-enriched transcription factor HNF-1α in expression of the CYP2E1 gene. DNA Cell Biol.

[25] Ueno T, Gonzalez FJ (1990). Transcriptional control of the rat hepatic CYP2E1 gene. Mol Cell Biol.

[26] Pearson C, Bartell T, Wang G, Hong X, Rusk SR, Fu L, Cerda S, Bustamante-Helfrich B, Kuohung W, Yarrington C, Adams WG, Wang X (2022). Birth cohort profile: rationale and study design. Precis Nutr.

[27] Roberts LD, Souza AL, Gerszten RE, Clish CB (2012). Targeted metabolomics. Curr Protoc Mol Biol.

[28] Aryee MJ, Jaffe AE, Corrada-Bravo H (2014). Minfi: a flexible and comprehensive bioconductor package for the analysis of Infinium DNA methylation microarrays. Bioinformatics.

[29] Fortin JP, Triche TJ, Hansen KD (2017). Preprocessing, normalization and integration of the Illumina HumanMethylationEPIC array with minfi. Bioinformatics.

[30] Bakulski KM, Feinberg JI, Andrews SV (2016). DNA methylation of cord blood cell types: applications for mixed cell birth studies. Epigenetics.

[31] Leek JT, Johnson WE, Parker HS, Jaffe AE, Storey JD (2012). The SVA package for removing batch effects and other unwanted variation in high-throughput experiments. Bioinformatics.

[32] Ritchie ME, Phipson B, Wu D (2015). limma powers differential expression analyses for RNA-sequencing and microarray studies. Nucleic Acids Res.

[33] Du P, Zhang X, Huang CC (2010). Comparison of Beta-value and M-value methods for quantifying methylation levels by microarray analysis. BMC Bioinform.

[34] Phipson B, Oshlack A (2014). DiffVar: a new method for detecting differential variability with application to methylation in cancer and aging. Genome Biol.

[35] Benjamini Y, Hochberg Y (1995). Controlling the false discovery rate: a practical and powerful approach to multiple testing. J R Stat Soc Ser B (Methodol).

[36] O’Leary NA, Wright MW, Brister JR (2016). Reference sequence (RefSeq) database at NCBI: current status, taxonomic expansion, and functional annotation. Nucleic Acids Res.

[37] Kent WJ, Sugnet CW, Furey TS (2002). The human genome browser at UCSC. Genome Res.

[38] Hahne F, Ivanek R (2016). Visualizing genomic data using Gviz and bioconductor. Methods Mol Biol.

