# Supplementary material for: Association between acetaminophen metabolites and CYP2E1 DNA methylation level in neonate cord blood in the Boston Birth Cohort
Source: Clin Epigenetics. 2023 Aug 18;15:132. doi: 10.1186/s13148-023-01551-4 (PMC10439592; doi:10.1186/s13148-023-01551-4)
Supplement: Supplementary file 1 — Additional file 1: Additional Tables 1–8. [file 13148_2023_1551_MOESM1_ESM.docx]

**Additional file 1: Figure 1. Histograms of the raw intensities of acetaminophen metabolites from** **liquid chromatography-tandem mass spectrometry (LC-MS).** The horizontal axis shows the raw intensity of each acetaminophen metabolite on the log_10_ scale. The vertical axis shows the frequency for the specific bin. For each metabolite, 100 bins were created across the range of the raw intensity from LC-MS measurement. Red dotted line shows the background noise level for each metabolite. Values that were detected by LC-MS but below the noise level were colored in gray. Values above the noise level were colored in black.


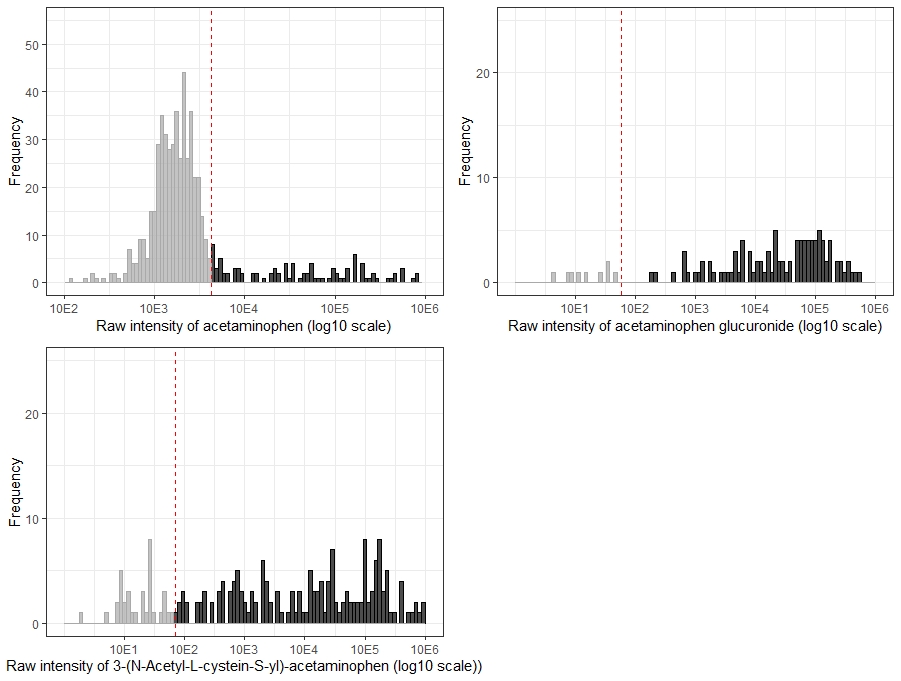


Background noise level: 43,000

Background noise level: 597

Background noise level: 700

Detection of acetaminophen metabolites by LC-MS


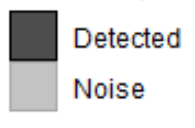


Detected

Not detected

| Additional file 1: Table 1. Results of adjusted linear regression models testing for differential methylation, at each of the 28 CpGs located in *CYP2E1*, between participants with (n=100) and without (n=470) acetaminophen glucuronide detection, sorted by P-value | | | | | | | | | |
| --- | --- | --- | --- | --- | --- | --- | --- | --- | --- |
| CpG site |  | **%Difference (95% CI) in DNAm: comparing detected group with not detected group** | | **P-value (robust)** | **FDR** | **Genomic coordinate (GRCh37/hg19)** | **Gene Name** | **Feature category ^a^** | **Relation to CpG Island** |
| cg23400446* |  | -4.32% | (-7.8%,-0.8%) | 2.34E-03 | 0.048 | chr10:135342560 | *CYP2E1* | Body | Island |
| cg05473257* |  | -4.76% | (-8.5%,-1%) | 3.81E-03 | 0.048 | chr10:135341443 | *CYP2E1* | Body | Island |
| cg19721068 |  | 1.07% | (0%,2.1%) | 6.30E-03 | 0.048 | chr10:135346592 | *CYP2E1* | Body | Open Sea |
| cg13315147* |  | -4.13% | (-7.6%,-0.7%) | 6.84E-03 | 0.048 | chr10:135341528 | *CYP2E1* | Body | Island |
| cg19469447* |  | -3.83% | (-7%,-0.7%) | 1.03E-02 | 0.053 | chr10:135341870 | *CYP2E1* | Body | Island |
| cg05194426* |  | -5.73% | (-10.4%,-1.1%) | 1.25E-02 | 0.053 | chr10:135343193 | *CYP2E1* | Body | South Shore |
| cg10862468* |  | -3.76% | (-6.9%,-0.7%) | 1.32E-02 | 0.053 | chr10:135342218 | *CYP2E1* | Body | Island |
| cg03134882 |  | -3.95% | (-7.3%,-0.6%) | 2.57E-02 | 0.081 | chr10:135341463 | *CYP2E1* | Body | Island |
| cg25330361* |  | -3.41% | (-6%,-0.8%) | 2.61E-02 | 0.081 | chr10:135342413 | *CYP2E1* | Body | Island |
| cg11445109 |  | -3.07% | (-6%,-0.2%) | 3.93E-02 | 0.110 | chr10:135343248 | *CYP2E1* | Body | South Shore |
| cg18984983 |  | -3.43% | (-6%,-0.9%) | 5.54E-02 | 0.131 | chr10:135342936 | *CYP2E1* | Body | South Shore |
| cg00321709 |  | -5.48% | (-9.8%,-1.2%) | 5.62E-02 | 0.131 | chr10:135341933 | *CYP2E1* | Body | Island |
| cg24530264 |  | -3.10% | (-5.5%,-0.7%) | 8.29E-02 | 0.179 | chr10:135342620 | *CYP2E1* | Body | South Shore |
| cg19571004 |  | -0.91% | (-2.1%,0.3%) | 1.11E-01 | 0.223 | chr10:135340850 | *CYP2E1* | TSS200 | North Shore |
| cg09208540 |  | -0.26% | (-0.7%,0.2%) | 1.41E-01 | 0.264 | chr10:135340467 | *CYP2E1* | TSS1500 | North Shore |
| cg00436603 |  | 0.78% | (-0.4%,1.9%) | 1.94E-01 | 0.339 | chr10:135340740 | *CYP2E1* | TSS200 | North Shore |
| cg10986462 |  | 0.17% | (-1.6%,2%) | 3.23E-01 | 0.533 | chr10:135340539 | *CYP2E1* | TSS1500 | North Shore |
| cg21024264 |  | -0.63% | (-1.9%,0.6%) | 4.04E-01 | 0.628 | chr10:135341025 | *CYP2E1* | 1stExon | North Shore |
| cg07381788 |  | -0.20% | (-0.8%,0.4%) | 4.39E-01 | 0.647 | chr10:135340445 | *CYP2E1* | TSS1500 | North Shore |
| cg26065573 |  | 0.38% | (-0.2%,1%) | 4.68E-01 | 0.655 | chr10:135339469 | *CYP2E1* | TSS1500 | North Shore |
| cg00720244 |  | 0.08% | (-0.3%,0.5%) | 5.82E-01 | 0.753 | chr10:135347434 | *CYP2E1* | Body | Open Sea |
| cg16538390 |  | -0.17% | (-0.8%,0.4%) | 5.96E-01 | 0.753 | chr10:135344917 | *CYP2E1* | Body | South Shelf |
| cg08472147 |  | 0.28% | (-0.7%,1.3%) | 6.19E-01 | 0.753 | chr10:135340583 | *CYP2E1* | TSS1500 | North Shore |
| cg05417377 |  | 0.03% | (-1%,1.1%) | 8.00E-01 | 0.934 | chr10:135350807 | *CYP2E1* | Body | Open Sea |
| cg14250048 |  | 0.01% | (-1%,1%) | 8.67E-01 | 0.966 | chr10:135340785 | *CYP2E1* | TSS200 | North Shore |
| cg01465364 |  | -0.06% | (-1.2%,1.1%) | 8.99E-01 | 0.966 | chr10:135340721 | *CYP2E1* | TSS200 | North Shore |
| cg01355198 |  | -0.01% | (-0.5%,0.5%) | 9.32E-01 | 0.966 | chr10:135347330 | *CYP2E1* | Body | Open Sea |
| cg19837601 |  | 0.06% | (-1.3%,1.4%) | 9.99E-01 | 0.999 | chr10:135340871 | *CYP2E1;CYP2E1* | 5'UTR;1stExon | North Shore |

Abbreviations: DNAm, DNA methylation; FDR, false discovery rate; chr, chromosome. Average DNAm and %Difference in DNAm were calculated from adjusted linear regression models with DNAm beta value as the dependent variable. P-value and FDR were obtained from adjusted linear regression models with DNAm M-value as the dependent variables. Covariates are child sex, delivery type, parity, gestational age, birthweight, maternal age, maternal marital status, prenatal smoking, prenatal alcohol use, intrauterine inflammation, preeclampsia, diabetes mellitus, maternal stress, estimated cell types, and 2 surrogate variables. ^a^ Gene feature category of CpG sites obtained from UCSC database: TSS, transcription start site; TSS200, 200 bases from TSS; TSS1500, 1500 bases from TSS; UTR, untranslated region. * CpG sites that were differentially methylated (FDR<0.05) with respect to detection of acetaminophen in the main analysis (table 2).

| Additional file 1: Table 2. Results of adjusted linear regression models testing for differential methylation, at each of the 28 CpGs located in *CYP2E1*, between participants with (n=165) and without (n=405) 3-(N-acetyl-L-cystein-S-yl)-acetaminophen detection, sorted by P-value | | | | | | | | | |
| --- | --- | --- | --- | --- | --- | --- | --- | --- | --- |
| CpG site |  | **%Difference (95% CI) in DNAm: comparing detected group with not detected group** | | **P-value (robust)** | **FDR** | **Genomic coordinate (GRCh37/hg19)** | **Gene Name** | **Feature category ^a^** | **Relation to CpG Island** |
| cg23400446* |  | -3.26% | (-6.1%,-0.4%) | 2.19E-03 | 0.047 | chr10:135342560 | *CYP2E1* | Body | Island |
| cg13315147* |  | -3.26% | (-6.1%,-0.4%) | 3.37E-03 | 0.047 | chr10:135341528 | *CYP2E1* | Body | Island |
| cg25330361* |  | -2.75% | (-4.9%,-0.6%) | 9.06E-03 | 0.049 | chr10:135342413 | *CYP2E1* | Body | Island |
| cg03134882 |  | -3.21% | (-5.9%,-0.5%) | 9.61E-03 | 0.049 | chr10:135341463 | *CYP2E1* | Body | Island |
| cg05194426* |  | -4.70% | (-8.5%,-0.9%) | 1.02E-02 | 0.049 | chr10:135343193 | *CYP2E1* | Body | South Shore |
| cg10862468* |  | -3.10% | (-5.6%,-0.6%) | 1.06E-02 | 0.049 | chr10:135342218 | *CYP2E1* | Body | Island |
| cg05473257* |  | -3.62% | (-6.7%,-0.5%) | 1.57E-02 | 0.063 | chr10:135341443 | *CYP2E1* | Body | Island |
| cg19469447* |  | -3.04% | (-5.7%,-0.4%) | 2.07E-02 | 0.073 | chr10:135341870 | *CYP2E1* | Body | Island |
| cg00321709 |  | -4.49% | (-8%,-0.9%) | 2.91E-02 | 0.090 | chr10:135341933 | *CYP2E1* | Body | Island |
| cg24530264 |  | -2.08% | (-4.1%,-0.1%) | 7.06E-02 | 0.198 | chr10:135342620 | *CYP2E1* | Body | South Shore |
| cg18984983 |  | -1.79% | (-3.9%,0.3%) | 1.14E-01 | 0.278 | chr10:135342936 | *CYP2E1* | Body | South Shore |
| cg21024264 |  | -1.13% | (-2.2%,-0.1%) | 1.19E-01 | 0.278 | chr10:135341025 | *CYP2E1* | 1stExon | North Shore |
| cg09208540 |  | -0.35% | (-0.7%,0%) | 1.55E-01 | 0.334 | chr10:135340467 | *CYP2E1* | TSS1500 | North Shore |
| cg19721068 |  | 0.30% | (-0.5%,1.1%) | 1.73E-01 | 0.338 | chr10:135346592 | *CYP2E1* | Body | Open Sea |
| cg11445109 |  | -2.04% | (-4.4%,0.4%) | 1.81E-01 | 0.338 | chr10:135343248 | *CYP2E1* | Body | South Shore |
| cg10986462 |  | -0.62% | (-2.1%,0.9%) | 2.12E-01 | 0.370 | chr10:135340539 | *CYP2E1* | TSS1500 | North Shore |
| cg19837601 |  | -0.97% | (-2.1%,0.1%) | 2.54E-01 | 0.419 | chr10:135340871 | *CYP2E1;CYP2E1* | 5'UTR;1stExon | North Shore |
| cg07381788 |  | -0.30% | (-0.8%,0.2%) | 3.97E-01 | 0.617 | chr10:135340445 | *CYP2E1* | TSS1500 | North Shore |
| cg01465364 |  | -0.65% | (-1.6%,0.3%) | 4.89E-01 | 0.703 | chr10:135340721 | *CYP2E1* | TSS200 | North Shore |
| cg19571004 |  | -0.70% | (-1.7%,0.3%) | 5.02E-01 | 0.703 | chr10:135340850 | *CYP2E1* | TSS200 | North Shore |
| cg01355198 |  | -0.16% | (-0.6%,0.2%) | 6.45E-01 | 0.860 | chr10:135347330 | *CYP2E1* | Body | Open Sea |
| cg14250048 |  | -0.32% | (-1.1%,0.5%) | 7.73E-01 | 0.942 | chr10:135340785 | *CYP2E1* | TSS200 | North Shore |
| cg00436603 |  | -0.21% | (-1.2%,0.8%) | 8.10E-01 | 0.942 | chr10:135340740 | *CYP2E1* | TSS200 | North Shore |
| cg00720244 |  | -0.06% | (-0.4%,0.3%) | 8.54E-01 | 0.942 | chr10:135347434 | *CYP2E1* | Body | Open Sea |
| cg16538390 |  | -0.03% | (-0.5%,0.5%) | 8.90E-01 | 0.942 | chr10:135344917 | *CYP2E1* | Body | South Shelf |
| cg08472147 |  | -0.03% | (-0.8%,0.8%) | 8.96E-01 | 0.942 | chr10:135340583 | *CYP2E1* | TSS1500 | North Shore |
| cg26065573 |  | 0.07% | (-0.4%,0.6%) | 9.08E-01 | 0.942 | chr10:135339469 | *CYP2E1* | TSS1500 | North Shore |
| cg05417377 |  | -0.12% | (-1%,0.8%) | 9.50E-01 | 0.950 | chr10:135350807 | *CYP2E1* | Body | Island |

Abbreviations: DNAm, DNA methylation; FDR, false discovery rate; chr, chromosome. Average DNAm and %Difference in DNAm were calculated from adjusted linear regression models with DNAm beta value as the dependent variable. P-value and FDR were obtained from adjusted linear regression models with DNAm M-value as the dependent variables. Covariates are child sex, delivery type, parity, gestational age, birthweight, maternal age, maternal marital status, prenatal smoking, prenatal alcohol use, intrauterine inflammation, preeclampsia, diabetes mellitus, maternal stress, estimated cell types, and 2 surrogate variables. ^a^ Gene feature category of CpG sites obtained from UCSC database: TSS, transcription start site; TSS200, 200 bases from TSS; TSS1500, 1500 bases from TSS; UTR, untranslated region. * CpG sites that were differentially methylated (FDR<0.05) with respect to detection of acetaminophen in the main analysis (table 2).

| Additional file 1: Table 3. Results of linear regression models testing for differential methylation level with further adjustment of race/ethnicity, at each of the 28 CpGs located in *CYP2E1*, between participants with (n=96) and without (n=474) acetaminophen detection, sorted by P-value | | | | | | | | | |
| --- | --- | --- | --- | --- | --- | --- | --- | --- | --- |
| CpG site |  | **%Difference (95% CI) in DNAm: comparing detected group with not detected group** | | **P-value (robust)** | **FDR** | **Genomic coordinate (GRCh37/hg19)** | **Gene Name** | **Feature category ^a^** | **Relation to CpG Island** |
| cg13315147* |  | -4.37% | (-7.9%,-0.8%) | 9.18E-04 | 0.026 | chr10:135341528 | *CYP2E1* | Body | Island |
| cg05473257* |  | -4.74% | (-8.6%,-0.9%) | 3.13E-03 | 0.044 | chr10:135341443 | *CYP2E1* | Body | Island |
| cg23400446* |  | -4.31% | (-7.9%,-0.7%) | 5.08E-03 | 0.047 | chr10:135342560 | *CYP2E1* | Body | Island |
| cg25330361* |  | -3.65% | (-6.4%,-0.9%) | 9.18E-03 | 0.059 | chr10:135342413 | *CYP2E1* | Body | Island |
| cg19469447* |  | -4.01% | (-7.3%,-0.7%) | 1.11E-02 | 0.059 | chr10:135341870 | *CYP2E1* | Body | Island |
| cg05194426* |  | -5.45% | (-10.3%,-0.6%) | 1.35E-02 | 0.059 | chr10:135343193 | *CYP2E1* | Body | South Shore |
| cg10862468* |  | -3.69% | (-6.9%,-0.5%) | 1.46E-02 | 0.059 | chr10:135342218 | *CYP2E1* | Body | Island |
| cg03134882 |  | -3.95% | (-7.4%,-0.5%) | 1.94E-02 | 0.068 | chr10:135341463 | *CYP2E1* | Body | Island |
| cg19571004 |  | -1.22% | (-2.5%,0%) | 2.66E-02 | 0.083 | chr10:135340850 | *CYP2E1* | TSS200 | North Shore |
| cg00321709 |  | -5.79% | (-10.3%,-1.3%) | 3.36E-02 | 0.094 | chr10:135341933 | *CYP2E1* | Body | Island |
| cg19721068 |  | 0.88% | (-0.2%,1.9%) | 4.79E-02 | 0.122 | chr10:135346592 | *CYP2E1* | Body | Open Sea |
| cg11445109 |  | -2.90% | (-5.9%,0.1%) | 6.02E-02 | 0.140 | chr10:135343248 | *CYP2E1* | Body | South Shore |
| cg18984983 |  | -3.23% | (-5.9%,-0.6%) | 9.68E-02 | 0.208 | chr10:135342936 | *CYP2E1* | Body | South Shore |
| cg24530264 |  | -3.13% | (-5.6%,-0.7%) | 1.17E-01 | 0.234 | chr10:135342620 | *CYP2E1* | Body | South Shore |
| cg26065573 |  | 0.47% | (-0.1%,1.1%) | 2.90E-01 | 0.542 | chr10:135339469 | *CYP2E1* | TSS1500 | North Shore |
| cg00436603 |  | 0.60% | (-0.6%,1.8%) | 3.72E-01 | 0.650 | chr10:135340740 | *CYP2E1* | TSS200 | North Shore |
| cg21024264 |  | -0.50% | (-1.8%,0.8%) | 4.59E-01 | 0.755 | chr10:135341025 | *CYP2E1* | 1stExon | North Shore |
| cg07381788 |  | -0.12% | (-0.7%,0.5%) | 4.95E-01 | 0.762 | chr10:135340445 | *CYP2E1* | TSS1500 | North Shore |
| cg01355198 |  | -0.08% | (-0.6%,0.4%) | 5.34E-01 | 0.762 | chr10:135347330 | *CYP2E1* | Body | Open Sea |
| cg09208540 |  | -0.13% | (-0.6%,0.3%) | 5.44E-01 | 0.762 | chr10:135340467 | *CYP2E1* | TSS1500 | North Shore |
| cg14250048 |  | 0.04% | (-1%,1%) | 6.99E-01 | 0.919 | chr10:135340785 | *CYP2E1* | TSS200 | North Shore |
| cg01465364 |  | -0.09% | (-1.3%,1.1%) | 7.32E-01 | 0.919 | chr10:135340721 | *CYP2E1* | TSS200 | North Shore |
| cg10986462 |  | 0.67% | (-1.2%,2.5%) | 7.71E-01 | 0.919 | chr10:135340539 | *CYP2E1* | TSS1500 | North Shore |
| cg16538390 |  | -0.09% | (-0.7%,0.5%) | 8.39E-01 | 0.919 | chr10:135344917 | *CYP2E1* | Body | South Shelf |
| cg05417377 |  | 0.02% | (-1.1%,1.1%) | 8.57E-01 | 0.919 | chr10:135350807 | *CYP2E1* | Body | Open Sea |
| cg19837601 |  | 0.15% | (-1.3%,1.5%) | 8.78E-01 | 0.919 | chr10:135340871 | *CYP2E1;CYP2E1* | 5'UTR;1stExon | North Shore |
| cg08472147 |  | 0.00% | (-1%,1%) | 8.86E-01 | 0.919 | chr10:135340583 | *CYP2E1* | TSS1500 | North Shore |
| cg00720244 |  | 0.06% | (-0.4%,0.5%) | 9.88E-01 | 0.988 | chr10:135347434 | *CYP2E1* | Body | Open Sea |

Abbreviations: DNAm, DNA methylation; FDR, false discovery rate; chr, chromosome. Average DNAm and %Difference in DNAm were calculated from adjusted linear regression models with DNAm beta value as the dependent variable. P-value and FDR were obtained from adjusted linear regression models with DNAm M-value as the dependent variables. Covariates are race/ethnicity, child sex, delivery type, parity, gestational age, birthweight, maternal age, maternal marital status, prenatal smoking, prenatal alcohol use, intrauterine inflammation, preeclampsia, diabetes mellitus, maternal stress, estimated cell types, and 2 surrogate variables. ^a^ Gene feature category of CpG sites obtained from UCSC database: TSS, transcription start site; TSS200, 200 bases from TSS; TSS1500, 1500 bases from TSS; UTR, untranslated region. * CpG sites that were differentially methylated (FDR<0.05) with respect to detection of acetaminophen in the main analysis (table 2).

| Additional file 1: Table 4. Results of adjusted linear regression models testing for differential methylation, at each of the 28 CpGs located in *CYP2E1*, between participants with (n=90) and without (n=441) acetaminophen detection and no maternal gestational diabetes, sorted by P-value | | | | | | | | | |
| --- | --- | --- | --- | --- | --- | --- | --- | --- | --- |
| CpG site |  | **%Difference (95% CI) in DNAm: comparing detected group with not detected group** | | **P-value (robust)** | **FDR** | **Genomic coordinate (GRCh37/hg19)** | **Gene Name** | **Feature category ^a^** | **Relation to CpG Island** |
| cg23400446* |  | -4.14% | (-7.9%,-0.4%) | 3.76E-03 | 0.078 | chr10:135342560 | *CYP2E1* | Body | Island |
| cg13315147* |  | -3.91% | (-7.6%,-0.3%) | 8.38E-03 | 0.078 | chr10:135341528 | *CYP2E1* | Body | Island |
| cg05473257* |  | -4.39% | (-8.4%,-0.4%) | 8.69E-03 | 0.078 | chr10:135341443 | *CYP2E1* | Body | Island |
| cg25330361* |  | -3.50% | (-6.3%,-0.7%) | 1.12E-02 | 0.078 | chr10:135342413 | *CYP2E1* | Body | Island |
| cg05194426* |  | -5.36% | (-10.3%,-0.4%) | 1.53E-02 | 0.086 | chr10:135343193 | *CYP2E1* | Body | South Shore |
| cg10862468* |  | -3.53% | (-6.8%,-0.2%) | 2.22E-02 | 0.101 | chr10:135342218 | *CYP2E1* | Body | Island |
| cg19469447* |  | -3.68% | (-7.1%,-0.3%) | 2.87E-02 | 0.101 | chr10:135341870 | *CYP2E1* | Body | Island |
| cg00321709 |  | -5.83% | (-10.4%,-1.2%) | 3.56E-02 | 0.101 | chr10:135341933 | *CYP2E1* | Body | Island |
| cg19571004 |  | -1.27% | (-2.6%,0.1%) | 3.60E-02 | 0.101 | chr10:135340850 | *CYP2E1* | TSS200 | North Shore |
| cg19721068 |  | 0.99% | (-0.1%,2.1%) | 3.61E-02 | 0.101 | chr10:135346592 | *CYP2E1* | Body | Open Sea |
| cg03134882 |  | -3.56% | (-7.1%,0%) | 5.10E-02 | 0.130 | chr10:135341463 | *CYP2E1* | Body | Island |
| cg24530264 |  | -3.05% | (-5.6%,-0.5%) | 1.08E-01 | 0.239 | chr10:135342620 | *CYP2E1* | Body | South Shore |
| cg11445109 |  | -2.69% | (-5.8%,0.4%) | 1.17E-01 | 0.239 | chr10:135343248 | *CYP2E1* | Body | South Shore |
| cg18984983 |  | -3.14% | (-5.9%,-0.4%) | 1.20E-01 | 0.239 | chr10:135342936 | *CYP2E1* | Body | South Shore |
| cg26065573 |  | 0.47% | (-0.2%,1.1%) | 2.83E-01 | 0.528 | chr10:135339469 | *CYP2E1* | TSS1500 | North Shore |
| cg00436603 |  | 0.59% | (-0.7%,1.8%) | 3.32E-01 | 0.582 | chr10:135340740 | *CYP2E1* | TSS200 | North Shore |
| cg01355198 |  | -0.13% | (-0.7%,0.4%) | 3.86E-01 | 0.636 | chr10:135347330 | *CYP2E1* | Body | Open Sea |
| cg09208540 |  | -0.18% | (-0.6%,0.3%) | 4.79E-01 | 0.746 | chr10:135340467 | *CYP2E1* | TSS1500 | North Shore |
| cg07381788 |  | -0.12% | (-0.8%,0.5%) | 5.86E-01 | 0.823 | chr10:135340445 | *CYP2E1* | TSS1500 | North Shore |
| cg05417377 |  | 0.18% | (-1%,1.3%) | 5.88E-01 | 0.823 | chr10:135350807 | *CYP2E1* | Body | Open Sea |
| cg21024264 |  | -0.37% | (-1.7%,1%) | 6.91E-01 | 0.875 | chr10:135341025 | *CYP2E1* | 1stExon | North Shore |
| cg19837601 |  | 0.20% | (-1.3%,1.7%) | 7.30E-01 | 0.875 | chr10:135340871 | *CYP2E1;CYP2E1* | 5'UTR;1stExon | North Shore |
| cg16538390 |  | -0.17% | (-0.8%,0.5%) | 7.45E-01 | 0.875 | chr10:135344917 | *CYP2E1* | Body | South Shelf |
| cg10986462 |  | 0.21% | (-1.6%,2%) | 7.54E-01 | 0.875 | chr10:135340539 | *CYP2E1* | TSS1500 | North Shore |
| cg01465364 |  | -0.11% | (-1.4%,1.1%) | 8.03E-01 | 0.875 | chr10:135340721 | *CYP2E1* | TSS200 | North Shore |
| cg14250048 |  | 0.07% | (-1%,1.1%) | 8.12E-01 | 0.875 | chr10:135340785 | *CYP2E1* | TSS200 | North Shore |
| cg08472147 |  | 0.12% | (-0.9%,1.2%) | 8.84E-01 | 0.916 | chr10:135340583 | *CYP2E1* | TSS1500 | North Shore |
| cg00720244 |  | 0.07% | (-0.4%,0.5%) | 9.77E-01 | 0.977 | chr10:135347434 | *CYP2E1* | Body | Open Sea |

Abbreviations: DNAm, DNA methylation; FDR, false discovery rate; chr, chromosome. Average DNAm and %Difference in DNAm were calculated from adjusted linear regression models with DNAm beta value as the dependent variable. P-value and FDR were obtained from adjusted linear regression models with DNAm M-value as the dependent variables. Covariates are child sex, delivery type, parity, gestational age, birthweight, maternal age, maternal marital status, prenatal smoking, prenatal alcohol use, intrauterine inflammation, preeclampsia, diabetes mellitus, maternal stress, estimated cell types, and 2 surrogate variables. ^a^ Gene feature category of CpG sites obtained from UCSC database: TSS, transcription start site; TSS200, 200 bases from TSS; TSS1500, 1500 bases from TSS; UTR, untranslated region. * CpG sites that were differentially methylated (FDR<0.05) with respect to detection of acetaminophen in the main analysis (table 2).

| Additional file 1: Table 5. Results of adjusted models testing for differences in methylation variation, at each of the 28 CpGs located in *CYP2E1*, between participants with (n=100) and without (n=470) acetaminophen glucuronide detection, sorted by P-value | | | | | | | | | | |
| --- | --- | --- | --- | --- | --- | --- | --- | --- | --- | --- |
| CpG site | **Sample variance** | **Log variance ratio** | **Difference in Levene Residuals** | | **P-value (robust)** | **FDR** | **Genomic coordinate (GRCh37/hg19)** | **Gene Name** | **Feature category ^a^** | **Relation to CpG Island** |
| cg18984983* | 1.004 | -0.394 | | -0.195 | 8.36E-05 | 0.001 | chr10:135342936 | *CYP2E1* | Body | South Shore |
| cg24530264* | 0.888 | -0.319 | | -0.185 | 1.06E-04 | 0.001 | chr10:135342620 | *CYP2E1* | Body | South Shore |
| cg03134882* | 0.805 | -0.190 | | -0.144 | 2.13E-04 | 0.002 | chr10:135341463 | *CYP2E1* | Body | Island |
| cg00321709* | 1.115 | -0.264 | | -0.188 | 7.24E-04 | 0.005 | chr10:135341933 | *CYP2E1* | Body | Island |
| cg13315147 | 0.818 | -0.187 | | -0.127 | 1.28E-03 | 0.007 | chr10:135341528 | *CYP2E1* | Body | Island |
| cg19469447* | 1.296 | -0.151 | | -0.133 | 5.08E-03 | 0.023 | chr10:135341870 | *CYP2E1* | Body | Island |
| cg05473257 | 1.327 | -0.151 | | -0.128 | 6.54E-03 | 0.023 | chr10:135341443 | *CYP2E1* | Body | Island |
| cg25330361 | 0.516 | -0.249 | | -0.122 | 6.67E-03 | 0.023 | chr10:135342413 | *CYP2E1* | Body | Island |
| cg21024264 | 0.139 | 0.326 | | 0.058 | 3.24E-02 | 0.101 | chr10:135341025 | *CYP2E1* | 1stExon | North Shore |
| cg23400446 | 1.423 | -0.093 | | -0.102 | 5.69E-02 | 0.159 | chr10:135342560 | *CYP2E1* | Body | Island |
| cg11445109 | 1.201 | -0.174 | | -0.093 | 1.94E-01 | 0.451 | chr10:135343248 | *CYP2E1* | Body | South Shore |
| cg10862468 | 0.578 | -0.044 | | -0.053 | 1.98E-01 | 0.451 | chr10:135342218 | *CYP2E1* | Body | Island |
| cg26065573 | 0.081 | -0.176 | | -0.024 | 2.09E-01 | 0.451 | chr10:135339469 | *CYP2E1* | TSS1500 | North Shore |
| cg05194426 | 1.161 | -0.075 | | -0.065 | 2.68E-01 | 0.533 | chr10:135343193 | *CYP2E1* | Body | South Shore |
| cg05417377 | 0.074 | 0.142 | | 0.020 | 2.85E-01 | 0.533 | chr10:135350807 | *CYP2E1* | Body | Open Sea |
| cg10986462 | 0.241 | -0.014 | | -0.043 | 3.55E-01 | 0.622 | chr10:135340539 | *CYP2E1* | TSS1500 | North Shore |
| cg19837601 | 0.104 | -0.053 | | 0.012 | 6.28E-01 | 0.875 | chr10:135340871 | *CYP2E1;CYP2E1* | 5'UTR;1stExon | North Shore |
| cg14250048 | 0.115 | -0.051 | | 0.012 | 6.30E-01 | 0.875 | chr10:135340785 | *CYP2E1* | TSS200 | North Shore |
| cg01465364 | 0.113 | 0.050 | | 0.009 | 7.15E-01 | 0.875 | chr10:135340721 | *CYP2E1* | TSS200 | North Shore |
| cg00720244 | 0.063 | 0.068 | | 0.006 | 7.28E-01 | 0.875 | chr10:135347434 | *CYP2E1* | Body | Open Sea |
| cg08472147 | 0.046 | 0.017 | | -0.005 | 7.49E-01 | 0.875 | chr10:135340583 | *CYP2E1* | TSS1500 | North Shore |
| cg00436603 | 0.104 | 0.038 | | 0.008 | 7.56E-01 | 0.875 | chr10:135340740 | *CYP2E1* | TSS200 | North Shore |
| cg07381788 | 0.063 | -0.014 | | -0.006 | 7.57E-01 | 0.875 | chr10:135340445 | *CYP2E1* | TSS1500 | North Shore |
| cg16538390 | 0.038 | 0.039 | | -0.004 | 7.57E-01 | 0.875 | chr10:135344917 | *CYP2E1* | Body | South Shelf |
| cg19571004 | 0.162 | 0.138 | | 0.008 | 7.81E-01 | 0.875 | chr10:135340850 | *CYP2E1* | TSS200 | North Shore |
| cg01355198 | 0.065 | 0.086 | | -0.004 | 8.45E-01 | 0.910 | chr10:135347330 | *CYP2E1* | Body | Open Sea |
| cg19721068 | 0.107 | -0.301 | | 0.004 | 8.90E-01 | 0.923 | chr10:135346592 | *CYP2E1* | Body | Open Sea |
| cg09208540 | 0.034 | -0.098 | | -0.001 | 9.39E-01 | 0.939 | chr10:135340467 | *CYP2E1* | TSS1500 | North Shore |

Abbreviations: DNAm, DNA methylation; FDR, false discovery rate; chr, chromosome. Covariates are child sex, delivery type, parity, gestational age, birthweight, maternal age, maternal marital status, prenatal smoking, prenatal alcohol use, intrauterine inflammation, preeclampsia, diabetes mellitus, maternal stress, estimated cell types, and 2 surrogate variables. ^a^ Gene feature category of CpG sites obtained from UCSC database: TSS, transcription start site; TSS200, 200 bases from TSS; TSS1500, 1500 bases from TSS; UTR, untranslated region. * CpG sites that were variably methylated (FDR<0.05) with respect to detection of acetaminophen in the main analysis (table 3).

| Additional file 1: Table 6. Results of adjusted models testing for differences in methylation variation, at each of the 28 CpGs located in *CYP2E1*, between participants with (n=165) and without (n=405) 3-(N-acetyl-L-cystein-S-yl)-acetaminophen detection, sorted by P-value | | | | | | | | | | |
| --- | --- | --- | --- | --- | --- | --- | --- | --- | --- | --- |
| CpG site | **Sample variance** | **Log variance ratio** | **Difference in Levene Residuals** | | **P-value (robust)** | **FDR** | **Genomic coordinate (GRCh37/hg19)** | **Gene Name** | **Feature category ^a^** | **Relation to CpG Island** |
| cg00321709* | 1.115 | -0.114 | | -0.124 | 7.05E-03 | 0.116 | chr10:135341933 | *CYP2E1* | Body | Island |
| cg24530264* | 0.888 | -0.114 | | -0.102 | 1.02E-02 | 0.116 | chr10:135342620 | *CYP2E1* | Body | South Shore |
| cg19469447* | 1.296 | -0.105 | | -0.098 | 1.24E-02 | 0.116 | chr10:135341870 | *CYP2E1* | Body | Island |
| cg03134882* | 0.805 | -0.068 | | -0.076 | 1.79E-02 | 0.125 | chr10:135341463 | *CYP2E1* | Body | Island |
| cg05473257 | 1.327 | -0.080 | | -0.087 | 2.68E-02 | 0.145 | chr10:135341443 | *CYP2E1* | Body | Island |
| cg25330361 | 0.516 | -0.103 | | -0.077 | 3.60E-02 | 0.145 | chr10:135342413 | *CYP2E1* | Body | Island |
| cg19837601 | 0.104 | 0.621 | | 0.043 | 3.64E-02 | 0.145 | chr10:135340871 | *CYP2E1;CYP2E1* | 5'UTR;1stExon | North Shore |
| cg18984983* | 1.004 | -0.092 | | -0.081 | 4.99E-02 | 0.175 | chr10:135342936 | *CYP2E1* | Body | South Shore |
| cg09208540 | 0.034 | 0.380 | | 0.017 | 1.36E-01 | 0.424 | chr10:135340467 | *CYP2E1* | TSS1500 | North Shore |
| cg21024264 | 0.139 | 0.469 | | 0.030 | 1.75E-01 | 0.460 | chr10:135341025 | *CYP2E1* | 1stExon | North Shore |
| cg13315147 | 0.818 | -0.012 | | -0.044 | 1.81E-01 | 0.460 | chr10:135341528 | *CYP2E1* | Body | Island |
| cg05417377 | 0.074 | 0.184 | | 0.019 | 2.19E-01 | 0.511 | chr10:135350807 | *CYP2E1* | Body | Open Sea |
| cg19571004 | 0.162 | 0.406 | | 0.027 | 2.70E-01 | 0.541 | chr10:135340850 | *CYP2E1* | TSS200 | North Shore |
| cg01465364 | 0.113 | 0.417 | | 0.023 | 2.71E-01 | 0.541 | chr10:135340721 | *CYP2E1* | TSS200 | North Shore |
| cg23400446 | 1.423 | 0.020 | | -0.044 | 3.18E-01 | 0.552 | chr10:135342560 | *CYP2E1* | Body | Island |
| cg11445109 | 1.201 | -0.081 | | -0.057 | 3.29E-01 | 0.552 | chr10:135343248 | *CYP2E1* | Body | South Shore |
| cg10862468 | 0.578 | 0.000 | | -0.033 | 3.37E-01 | 0.552 | chr10:135342218 | *CYP2E1* | Body | Island |
| cg00720244 | 0.063 | 0.092 | | 0.013 | 3.55E-01 | 0.552 | chr10:135347434 | *CYP2E1* | Body | Open Sea |
| cg14250048 | 0.115 | 0.421 | | 0.018 | 3.81E-01 | 0.561 | chr10:135340785 | *CYP2E1* | TSS200 | North Shore |
| cg19721068 | 0.107 | 0.138 | | 0.016 | 4.47E-01 | 0.625 | chr10:135346592 | *CYP2E1* | Body | Open Sea |
| cg00436603 | 0.104 | 0.482 | | 0.015 | 4.69E-01 | 0.625 | chr10:135340740 | *CYP2E1* | TSS200 | North Shore |
| cg16538390 | 0.038 | -0.008 | | -0.006 | 5.91E-01 | 0.752 | chr10:135344917 | *CYP2E1* | Body | South Shelf |
| cg07381788 | 0.063 | 0.189 | | -0.007 | 6.20E-01 | 0.755 | chr10:135340445 | *CYP2E1* | TSS1500 | North Shore |
| cg08472147 | 0.046 | 0.229 | | 0.006 | 6.58E-01 | 0.768 | chr10:135340583 | *CYP2E1* | TSS1500 | North Shore |
| cg05194426 | 1.161 | 0.064 | | -0.007 | 8.90E-01 | 0.993 | chr10:135343193 | *CYP2E1* | Body | South Shore |
| cg01355198 | 0.065 | 0.157 | | -0.001 | 9.43E-01 | 0.993 | chr10:135347330 | *CYP2E1* | Body | Open Sea |
| cg26065573 | 0.081 | 0.057 | | 0.000 | 9.80E-01 | 0.993 | chr10:135339469 | *CYP2E1* | TSS1500 | North Shore |
| cg10986462 | 0.241 | 0.327 | | 0.000 | 9.93E-01 | 0.993 | chr10:135340539 | *CYP2E1* | TSS1500 | North Shore |

Abbreviations: DNAm, DNA methylation; FDR, false discovery rate; chr, chromosome. Covariates are child sex, delivery type, parity, gestational age, birthweight, maternal age, maternal marital status, prenatal smoking, prenatal alcohol use, intrauterine inflammation, preeclampsia, diabetes mellitus, maternal stress, estimated cell types, and 2 surrogate variables. ^a^ Gene feature category of CpG sites obtained from UCSC database: TSS, transcription start site; TSS200, 200 bases from TSS; TSS1500, 1500 bases from TSS; UTR, untranslated region. * CpG sites that were variably methylated (FDR<0.05) with respect to detection of acetaminophen in the main analysis (table 3).

| Additional file 1: Table 7. Results of adjusted models testing for differences in methylation variation with further adjustment of race/ethnicity, at each of the 28 CpGs located in *CYP2E1*, between participants with (n=96) and without (n=474) acetaminophen detection, sorted by P-value | | | | | | | | | | |
| --- | --- | --- | --- | --- | --- | --- | --- | --- | --- | --- |
| CpG site | **Sample variance** | **Log variance ratio** | **Difference in Levene Residuals** | | **P-value (robust)** | **FDR** | **Genomic coordinate (GRCh37/hg19)** | **Gene Name** | **Feature category ^a^** | **Relation to CpG Island** |
| cg24530264* | 0.888 | -0.303 | | -0.178 | 3.18E-04 | 0.009 | chr10:135342620 | *CYP2E1* | Body | South Shore |
| cg18984983* | 1.004 | -0.364 | | -0.166 | 1.23E-03 | 0.013 | chr10:135342936 | *CYP2E1* | Body | South Shore |
| cg00321709* | 1.115 | -0.260 | | -0.183 | 1.44E-03 | 0.013 | chr10:135341933 | *CYP2E1* | Body | Island |
| cg03134882* | 0.805 | -0.145 | | -0.124 | 2.03E-03 | 0.014 | chr10:135341463 | *CYP2E1* | Body | Island |
| cg19469447* | 1.296 | -0.142 | | -0.144 | 3.24E-03 | 0.018 | chr10:135341870 | *CYP2E1* | Body | Island |
| cg13315147 | 0.818 | -0.119 | | -0.093 | 2.27E-02 | 0.106 | chr10:135341528 | *CYP2E1* | Body | Island |
| cg05473257 | 1.327 | -0.116 | | -0.106 | 3.05E-02 | 0.122 | chr10:135341443 | *CYP2E1* | Body | Island |
| cg25330361 | 0.516 | -0.214 | | -0.096 | 3.81E-02 | 0.133 | chr10:135342413 | *CYP2E1* | Body | Island |
| cg23400446 | 1.423 | -0.090 | | -0.092 | 9.64E-02 | 0.300 | chr10:135342560 | *CYP2E1* | Body | Island |
| cg21024264 | 0.139 | 0.245 | | 0.038 | 1.77E-01 | 0.467 | chr10:135341025 | *CYP2E1* | 1stExon | North Shore |
| cg26065573 | 0.081 | -0.241 | | -0.027 | 1.84E-01 | 0.467 | chr10:135339469 | *CYP2E1* | TSS1500 | North Shore |
| cg09208540 | 0.034 | 0.162 | | 0.018 | 2.00E-01 | 0.467 | chr10:135340467 | *CYP2E1* | TSS1500 | North Shore |
| cg07381788 | 0.063 | -0.121 | | -0.023 | 2.29E-01 | 0.493 | chr10:135340445 | *CYP2E1* | TSS1500 | North Shore |
| cg11445109 | 1.201 | -0.144 | | -0.075 | 3.05E-01 | 0.590 | chr10:135343248 | *CYP2E1* | Body | South Shore |
| cg10862468 | 0.578 | 0.016 | | -0.043 | 3.18E-01 | 0.590 | chr10:135342218 | *CYP2E1* | Body | Island |
| cg10986462 | 0.241 | 0.068 | | -0.044 | 3.58E-01 | 0.590 | chr10:135340539 | *CYP2E1* | TSS1500 | North Shore |
| cg19837601 | 0.104 | 0.031 | | 0.023 | 3.65E-01 | 0.590 | chr10:135340871 | *CYP2E1;CYP2E1* | 5'UTR;1stExon | North Shore |
| cg08472147 | 0.046 | -0.070 | | -0.014 | 3.79E-01 | 0.590 | chr10:135340583 | *CYP2E1* | TSS1500 | North Shore |
| cg01355198 | 0.065 | -0.120 | | -0.016 | 4.03E-01 | 0.594 | chr10:135347330 | *CYP2E1* | Body | Open Sea |
| cg05417377 | 0.074 | 0.100 | | 0.014 | 4.78E-01 | 0.669 | chr10:135350807 | *CYP2E1* | Body | Open Sea |
| cg00720244 | 0.063 | -0.114 | | -0.011 | 5.53E-01 | 0.737 | chr10:135347434 | *CYP2E1* | Body | Open Sea |
| cg00436603 | 0.104 | -0.019 | | -0.011 | 6.56E-01 | 0.835 | chr10:135340740 | *CYP2E1* | TSS200 | North Shore |
| cg19721068 | 0.107 | -0.440 | | -0.010 | 7.20E-01 | 0.867 | chr10:135346592 | *CYP2E1* | Body | Open Sea |
| cg19571004 | 0.162 | 0.076 | | -0.010 | 7.43E-01 | 0.867 | chr10:135340850 | *CYP2E1* | TSS200 | North Shore |
| cg01465364 | 0.113 | -0.006 | | -0.005 | 8.45E-01 | 0.946 | chr10:135340721 | *CYP2E1* | TSS200 | North Shore |
| cg14250048 | 0.115 | -0.225 | | 0.004 | 8.78E-01 | 0.946 | chr10:135340785 | *CYP2E1* | TSS200 | North Shore |
| cg05194426 | 1.161 | 0.023 | | -0.006 | 9.23E-01 | 0.957 | chr10:135343193 | *CYP2E1* | Body | South Shore |
| cg16538390 | 0.038 | 0.098 | | 0.000 | 9.81E-01 | 0.981 | chr10:135344917 | *CYP2E1* | Body | South Shelf |

Abbreviations: DNAm, DNA methylation; FDR, false discovery rate; chr, chromosome. Covariates are race/ethnicity, child sex, delivery type, parity, gestational age, birthweight, maternal age, maternal marital status, prenatal smoking, prenatal alcohol use, intrauterine inflammation, preeclampsia, diabetes mellitus, maternal stress, estimated cell types, and 2 surrogate variables. ^a^ Gene feature category of CpG sites obtained from UCSC database: TSS, transcription start site; TSS200, 200 bases from TSS; TSS1500, 1500 bases from TSS; UTR, untranslated region. * CpG sites that were variably methylated (FDR<0.05) with respect to detection of acetaminophen in the main analysis (table 3).

| Additional file 1: Table 8. Results of adjusted models testing for differences in methylation variation, at each of the 28 CpGs located in *CYP2E1*, between participants with (n=90) and without (n=441) acetaminophen detection and no maternal gestational diabetes, sorted by P-value | | | | | | | | | | |
| --- | --- | --- | --- | --- | --- | --- | --- | --- | --- | --- |
| CpG site | **Sample variance** | **Log variance ratio** | **Difference in Levene Residuals** | | **P-value (robust)** | **FDR** | **Genomic coordinate (GRCh37/hg19)** | **Gene Name** | **Feature category ^a^** | **Relation to CpG Island** |
| cg24530264* | 0.880 | -0.304 | | -0.167 | 1.22E-03 | 0.016 | chr10:135342620 | *CYP2E1* | Body | South Shore |
| cg00321709* | 1.107 | -0.296 | | -0.195 | 1.22E-03 | 0.016 | chr10:135341933 | *CYP2E1* | Body | Island |
| cg18984983* | 1.003 | -0.362 | | -0.169 | 1.76E-03 | 0.016 | chr10:135342936 | *CYP2E1* | Body | South Shore |
| cg03134882* | 0.802 | -0.146 | | -0.128 | 2.57E-03 | 0.018 | chr10:135341463 | *CYP2E1* | Body | Island |
| cg19469447* | 1.287 | -0.148 | | -0.139 | 7.01E-03 | 0.039 | chr10:135341870 | *CYP2E1* | Body | Island |
| cg13315147 | 0.808 | -0.154 | | -0.109 | 1.11E-02 | 0.052 | chr10:135341528 | *CYP2E1* | Body | Island |
| cg05473257 | 1.315 | -0.110 | | -0.102 | 4.86E-02 | 0.194 | chr10:135341443 | *CYP2E1* | Body | Island |
| cg25330361 | 0.511 | -0.208 | | -0.084 | 7.80E-02 | 0.273 | chr10:135342413 | *CYP2E1* | Body | Island |
| cg09208540 | 0.035 | 0.198 | | 0.022 | 1.43E-01 | 0.421 | chr10:135340467 | *CYP2E1* | TSS1500 | North Shore |
| cg11445109 | 1.179 | -0.256 | | -0.109 | 1.50E-01 | 0.421 | chr10:135343248 | *CYP2E1* | Body | South Shore |
| cg23400446 | 1.408 | -0.077 | | -0.080 | 1.67E-01 | 0.424 | chr10:135342560 | *CYP2E1* | Body | Island |
| cg21024264 | 0.142 | 0.238 | | 0.039 | 1.82E-01 | 0.424 | chr10:135341025 | *CYP2E1* | 1stExon | North Shore |
| cg01355198 | 0.066 | -0.209 | | -0.024 | 2.23E-01 | 0.480 | chr10:135347330 | *CYP2E1* | Body | Open Sea |
| cg07381788 | 0.064 | -0.121 | | -0.023 | 2.46E-01 | 0.492 | chr10:135340445 | *CYP2E1* | TSS1500 | North Shore |
| cg26065573 | 0.083 | -0.241 | | -0.023 | 2.68E-01 | 0.499 | chr10:135339469 | *CYP2E1* | TSS1500 | North Shore |
| cg19837601 | 0.108 | 0.040 | | 0.028 | 2.98E-01 | 0.499 | chr10:135340871 | *CYP2E1;CYP2E1* | 5'UTR;1stExon | North Shore |
| cg05417377 | 0.074 | 0.077 | | 0.021 | 3.03E-01 | 0.499 | chr10:135350807 | *CYP2E1* | Body | Open Sea |
| cg10862468 | 0.571 | -0.003 | | -0.039 | 3.80E-01 | 0.590 | chr10:135342218 | *CYP2E1* | Body | Island |
| cg00720244 | 0.064 | -0.161 | | -0.012 | 5.28E-01 | 0.772 | chr10:135347434 | *CYP2E1* | Body | Open Sea |
| cg08472147 | 0.047 | -0.142 | | -0.010 | 5.67E-01 | 0.772 | chr10:135340583 | *CYP2E1* | TSS1500 | North Shore |
| cg19721068 | 0.110 | -0.551 | | -0.016 | 5.79E-01 | 0.772 | chr10:135346592 | *CYP2E1* | Body | Open Sea |
| cg16538390 | 0.039 | 0.145 | | 0.006 | 6.86E-01 | 0.873 | chr10:135344917 | *CYP2E1* | Body | South Shelf |
| cg10986462 | 0.221 | 0.238 | | -0.016 | 7.33E-01 | 0.892 | chr10:135340539 | *CYP2E1* | TSS1500 | North Shore |
| cg01465364 | 0.116 | 0.018 | | 0.006 | 8.14E-01 | 0.950 | chr10:135340721 | *CYP2E1* | TSS200 | North Shore |
| cg19571004 | 0.165 | 0.067 | | -0.004 | 8.95E-01 | 0.991 | chr10:135340850 | *CYP2E1* | TSS200 | North Shore |
| cg05194426 | 1.156 | -0.006 | | -0.005 | 9.31E-01 | 0.991 | chr10:135343193 | *CYP2E1* | Body | South Shore |
| cg14250048 | 0.116 | -0.405 | | 0.000 | 9.86E-01 | 0.991 | chr10:135340785 | *CYP2E1* | TSS200 | North Shore |
| cg00436603 | 0.107 | -0.010 | | 0.000 | 9.91E-01 | 0.991 | chr10:135340740 | *CYP2E1* | TSS200 | North Shore |

Abbreviations: DNAm, DNA methylation; FDR, false discovery rate; chr, chromosome. Covariates are child sex, delivery type, parity, gestational age, birthweight, maternal age, maternal marital status, prenatal smoking, prenatal alcohol use, intrauterine inflammation, preeclampsia, diabetes mellitus, maternal stress, estimated cell types, and 2 surrogate variables. ^a^ Gene feature category of CpG sites obtained from UCSC database: TSS, transcription start site; TSS200, 200 bases from TSS; TSS1500, 1500 bases from TSS; UTR, untranslated region. * CpG sites that were variably methylated (FDR<0.05) with respect to detection of acetaminophen in the main analysis (table 3).
